# Ligand-induced structural transitions combined with paramagnetic ions facilitate unambiguous NMR assignments of methyl groups in large proteins

**DOI:** 10.1007/s10858-022-00394-0

**Published:** 2022-04-10

**Authors:** Lars Mühlberg, Tuncay Alarcin, Thorben Maass, Robert Creutznacher, Richard Küchler, Alvaro Mallagaray

**Affiliations:** Institute for Chemistry and Metabolomics, Centre for Structural and Cell Biology in Medicine, University of Lübeck, Ratzeburger Allee 160, 23562 Lübeck, Germany

**Keywords:** MILVAT labelling, 4D HMQC-NOESY-HMQC, Paramagnetic NMR, UDP-glucose pyrophosphorylase from *Leishmania major*, Methyl-TROSY, Complete assignment

## Abstract

NMR spectroscopy allows the study of biomolecules in close-to-native conditions. Structural information can be inferred from the NMR spectra when an assignment is available. Protein assignment is usually a time-consuming task, being specially challenging in the case of large, supramolecular systems. Here, we present an extension of existing state-of-the-art strategies for methyl group assignment that partially overcomes signal overlapping and other difficulties associated to isolated methyl groups. Our approach exploits the ability of proteins to populate two or more conformational states, allowing for unique NOE restraints in each protein conformer. The method is compatible with automated assignment algorithms, granting assignments beyond the limits of a single protein state. The approach also benefits from long-range structural restraints obtained from metal-induced pseudocontact shifts (PCS) and paramagnetic relaxation enhancements (PREs). We illustrate the method with the complete assignment of the 199 methyl groups of a MIL^proS^V^proS^AT methyl-labeled sample of the UDP-glucose pyrophosphorylase enzyme from *Leishmania major* (LmUGP). Protozoan parasites of the genus *Leishmania* causes Leishmaniasis, a neglected disease affecting over 12 million people worldwide. LmUGP is responsible for the de novo biosynthesis of uridine diphosphate-glucose, a precursor in the biosynthesis of the dense surface glycocalyx involved in parasite survival and infectivity. NMR experiments with LmUGP and related enzymes have the potential to unravel new insights in the host resistance mechanisms used by *Leishmania major*. Our efforts will help in the development of selective and efficient drugs against *Leishmania*.

**Supplementary Information:**

The online version contains supplementary material available at 10.1007/s10858-022-00394-0.

## Introduction

NMR spectroscopy enables the study of the structure, dynamics and interactions of biomolecules in close-to-native conditions (Williamson 2013; Baldwin and Kay 2009; Bax and Clore 2019; Barrett et al. 2013). Solution NMR has classically made used of ^15^N isotopic labeling of amido groups in the protein backbone. However, the assignment of protein backbone resonances becomes challenging in large, supramolecular systems. The advent of ^1^H–^13^C methyl-transverse relaxation optimized spectroscopy (methyl TROSY) techniques (Tugarinov et al. 2003) offered a new avenue to overcome previous limitations, allowing solution NMR with protein ensembles as large as 1 MDa (Ruschak and Kay 2012; Rosenzweig and Kay 2014; Mas et al. 2018; Tugarinov et al. 2007; Kay 2011; Sprangers and Kay 2007; Gauto et al. 2019; Rosenzweig et al. 2015; Pederson et al. 2017; Shiraishi et al. 2018). Such an approach requires selective [^1^H,^13^C]-labeling of amino acid methyl groups in an otherwise highly deuterated background. Several protocols for efficient methyl labeling from *E. coli*, yeast, insect and mammalian cells have been developed. Broadly speaking, labeling is achieved by the addition of selectively labeled amino acid precursors (Ile, Leu, Val, Met) or by supplementation with selectively labeled amino acids (Ala, Met, Thr) (Schütz and Sprangers 2019).

Interpretation of methyl TROSY spectra benefits from a confident assignment of methyl group signals. When backbone assignments are available from triple-resonance experiments, pulse sequences transferring magnetization through scalar (Kay et al. 1990) or dipolar (Tugarinov and Kay 2003) couplings from backbone amides or carbonyls to methyl groups can be applied. Systematic mutagenesis provides an alternative path when a backbone signal assignment is not available (Amero et al. 2011). However, mutagenesis can be costly and time consuming, and mutations can perturb the chemical environment of the methyl groups in unforeseen ways. Therefore, this approach is currently limited to large, multi-domain ensembles, or to methyl groups which cannot be assigned by other means (Sprangers and Kay 2007; Velyvis et al. 2009). Recently, structure-based assignment strategies have emerged as a powerful alternative in the cases where high-resolution crystal structures are available. In general, such methods correlate NOE-derived distance restraints from 3D or 4D HMQC-NOESY experiments (Tugarinov et al. 2005; Wen et al. 2012) with structural data to produce consistent assignments of the methyl resonances. Complementary, paramagnetic relaxation enhancements (PREs) and pseudocontact shifts (PCS) can be used as a source of long-range information, validating or even expanding NOE-based assignments (Velyvis et al. 2009). Following this line of thoughts several automated algorithms have been developed for methyl group assignment (Pritišanac et al. 2020). They can be classified in three categories: Those that exclusively rely on inter-methyl NOEs [(*MAGIC* (Monneau et al. 2017), *MAGMA* (Pritisanac et al. 2017), *MAUS* (Nerli et al. 2021) and *MethylFLYA* (Pritisanac et al. 2019)], those which solely depend on PCS [*Possum* (John et al. 2007) and *PARAssign* (Lescanne et al. 2017; Skinner et al. 2013)] and mixed approaches [*MAP-XSII* (Xu and Matthews 2013), *PRE-ASSIGN* (Venditti et al. 2011) and *FLAMEnGO2.*0 (Chao et al. 2014)].

It has been recently shown that high probe density labeling schemes facilitate unambiguous NMR assignments of methyl groups (Proudfoot et al. 2016) Simultaneous labeling of all methyl-containing amino acids offers the highest probe density, but at the price of peak overlapping in large protein complexes. This problem can be partially overcome by comparing spectra of two different protein states (i.e., apo and holo in enzymes). The presence of a ligand bound to the protein alters the chemical environment of the surrounding atoms, inducing chemical shift perturbations (CSPs) Williamson (2013) CSPs are likely more pronounced if ligand binding stabilizes an alternative protein conformational state. In this scenario, average distances between certain protein methyl groups differ between conformers. For instance, spatially isolated methyl groups in the one state may be part of densely populated protein parts in the other state, allowing the assignment of otherwise isolated methyl groups. Here we demonstrate how ligand-induced changes of protein states can be exploited to expand currently available strategies for structural-based assignment of methyl groups. Our approach has three prerequisites: (1) Apo and bound states exist in fast or close-to-intermedium exchange in the NMR time scale, allowing transfer of the assignment between conformations. This can often be observed for protein–ligand interactions with dissociation constants *K*_D_s in the µM to mM range. (2) High-resolution crystal structures are available for both protein states. (3) Short- and/or long-range NMR structural information is available or can be extracted for both protein states (i.e. 3D or 4D NOESY-HMQC spectra, PCS, PREs…).

**Fig. 1:**
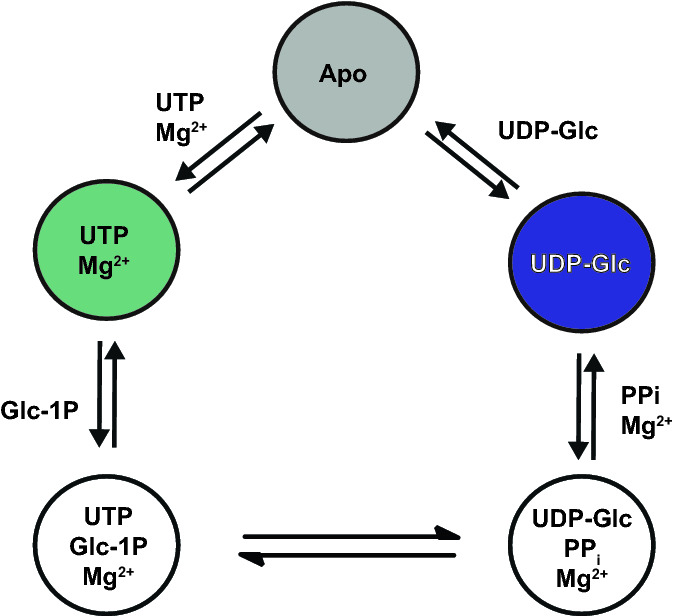
Catalytic cycle postulated for LmUGP. Protein states used for the assignment are apo, UTP:Mg^2+^ and UDP-Glc bound states (grey, green and blue, respectively)

In this work we have followed a structure-based assignment exploiting multiple ligand-induced protein states to obtain a complete assignment of Ala-β, Ile-γ1, Leu-δ2, Val-γ2, Met-ε and Thr-γ methyl [^13^C,^1^H_3_]-methyl-labeled enzyme UDP-glucose pyrophosphorylase from *Leishmania major* (MIL^proS^V^proS^AT LmUGP). *L. major* causes severe diseases in humans and animals, with symptoms ranging from self-healing cutaneous lesions to fatal visceral forms (WHO 1984). The enzyme plays a central role in the life cycle of *L. major*, as it catalyzes the reversible conversion of uridine-5´-triphosphate (UTP) and glucose 1-phosphate (Glc-1-P) into uridine diphosphate-glucose (UDP-Glc) and inorganic pyrophosphate (PP_i_) in the presence of Mg^2+^ (Fig. 1) (Lamerz et al. 2006). UDP-Glc can be converted into UDP-galactose, which is used in the biosynthesis of a dense layer of glycoconjugates covering the parasite. Depletion of UDP-galactose pools is associated with parasite growth arrest and cell death in vitro, rendering LmUGP as an attractive target for drug development (Damerow 2015). Methyl-labeled LmUGP exists as a soluble monomeric protein with a molecular weight of 62 kDa. The enzyme follows a sequential bi-bi catalytic mechanism, the binding of UTP or UDP-Glc being the first step in the for- and backward catalytic reactions, respectively. Crystallographic studies and molecular dynamic (MD) simulations have shown that LmUGP undergoes significant conformational changes along the catalytic cycle (Steiner et al. 2007; Führing et al. 2013). Therefore, LmUGP constitutes an excellent model for the use of multiple protein states in the assignment of systems containing a high probe density.

## Material and methods

### Synthesis and purification of methyl labeled LmUGP

The gene encoding for UDP-glycopyrophosphorylase from *L. major* (EC 2.7.7.9) was subcloned into the pET-22b (Novagen) expression vector as described previously (Lamerz et al. 2006) and transformed into *Escherichia coli* BL21(DE3). Single-point mutants T96S, A145G, T172S, A183G, T226S, A291G, A345G, V413I, A419G, A454G, A470G, T492S and M495I were generated by site-directed mutagenesis (Eurofins Genomics), and confirmed by DNA sequencing (Table S1). Primers used for mutagenesis are listed in Table S1. Mutants were expressed and isotopically labeled as described below according to the substituted amino acid type. Prior purification, pellets were combined as follows: batch 1: A419G, V413I and M495I; batch 2: A291G and T172S; batch 3: A454G and T96S; batch 4: A470G and T226S; batch 5: A345G and T492S. A145G and A183G were purified separately. This approach delivers a single labeling pattern for each mutant in each sample, minimizing the number of protein purifications required.

[*U*-^15^ N,^2^H], [^13^C,^1^H_3_]-methyl labeled UDP-glycopyrophosphorylase from LmUGP was expressed following an adapted version from previously reported protocols (Proudfoot et al. 2016; Muller-Hermes et al. 2020). Briefly, *E. coli* BL21(DE3) containing the gene for LmUGP were grown in 20 ml of LB Lennox medium (Roth) until an optical density of 600 nm (OD_600_) > 2 was reached. Ampicillin (100 µg/mL) was used as selecting agent through the expression. Unless otherwise stated, bacteria were grown at 37 °C under shaking (220 rpm). Cells for inoculation of 10 mL M9^+^/D_2_O minimal medium with a starting OD_600_ of 0.1 were harvested by centrifugation, and excess of TB medium was removed. This starter culture allows *E. coli* to acclimate to D_2_O, and was grown overnight. In all M9^+^/D_2_O minimal media, 3 g/L of ^15^ N-amonium chloride (Deutero) and 3 g/L of deuterated ^12^C-glucose (1,2,3,4,5,6,6-d_7_, Deutero) were used as the principal nitrogen and carbon sources, respectively. Detailed recipes for culture media used in this study can be found in the supplementary material. The next morning 2 ml of the starter culture were spin-down, supernatant was removed and cells were transferred into 20 mL of freshly prepared M9^+^/D_2_O minimal medium. When an OD_600_ of 0.4 was reached, the culture volume was increased to 90 ml and cells were grown until an OD_600_ of 0.6–0.8 was reached. At this point, the temperature of the incubator was reduced to 16 °C and 10 mL of M9^+^/D_2_O minimal medium containing the desired labeled precursors and amino acids were added (Table S2). Protein expression was induced after 1 h using 1 mM isopropyl β-D-1-thiogalactopyranoside (IPTG). Cells were harvested by centrifugation when the maximal cell density was reached (OD_600_ 3.8–4.8) and stored at -20 °C.

LmUGP was purified as described previously (Lamerz et al. 2006) with the following modifications: Bacterial pellet containing the overexpressed LmUGP was resuspended in purification buffer (20 mM Tris pH 7.8, 300 mM NaCl and 5 mM 2-mercaptoethanol, Sigma-Aldrich) containing 5 mM imidazole. Enzyme inhibitors aprotinin and leupeptin (4 µg/g wet pellet each, Roth), lysozyme (0.25 mg/g wet pellet, Merck) and benzonase (2.5 U/g wet pellet, Novagen) were added to the suspension and cells were lysated using a Microfluidizer® (12,000 psi). Cell lysate was centrifuged at 5,000 g for 1 h, and the soluble lysate was passed through a 5 mL Ni–NTA agarose column (GE Healthcare). The column was then washed with 50 mL purification buffer containing 40 mM imidazole concentration, and LmUGP was eluted using 20 mL of purification buffer at 300 mM imidazole concentration. Fractions showing UV absorption at 280 nm were pooled together and loaded into a HiLoad 16/600 Superdex 200 pg size exclusion column (GE Healthcare). Purified LmUGP was eluted in purification buffer, the elution fractions were combined and LmUGP was concentrated with Amicon Ultra-4 Centrifugal Filter Units (Millipore, MWCO 10 kDa). Protein samples were stored at 4 °C in the presence of 2 mM tris(2-carboxyethyl)phosphine (TCEP).

### NMR sample preparation

Storage buffer was changed into the desired NMR buffer using 2 mL Zeba™ Spin Desalting Columns (Thermo Fischer Scientific). Buffer A was used for routine ^1^H-^13^C HMQC, methyl-methyl NOESY, TRACT experiments, titrations of uridine-5'-diphosphate-glucose (UDP-Glc, Sigma-Aldrich) and uridine-5'-triphosphate (UTP, Sigma-Aldrich) over MIL^proS^V^proS^AT LmUGP and contained: 20 mM Tris-d_11_ (Eurisotop) pH* 7.20, 75 mM NaCl, 2 mM TCEP-d_16_ (CIL), 0.1 mM 2,2-Dimethyl-2-silapentane-5-sulfonate-d_6_ (DSS-d_6_, Sigma-Aldrich) and 0.2 mM imidazole in D_2_O (Eurisotop, 99.96%). Measurements of metal-induced PRE and PCS, and titrations of divalent and trivalent metals over UTP were conducted in buffer B, which was composed of 20 mM Bis–Tris-d_19_ (Sigma-Aldrich) pH* 7.06, 75 mM NaCl, 2 mM TCEP-d_16_ and 0.1 mM DSS-d_6_ in D_2_O. Protein concentrations were determined after buffer exchange by UV absorbance at 280 nm with ε = 42,860 M^−1^ cm^−1^. The extinction coefficient ε was obtained via absolute concentration measurements of a LmUGP sample using amino acid analysis. A complete list of samples prepared can be found in Table S3.

Dissociation constants *K*_D_ of UTP in the absence and in the presence of saturating concentrations of metals (10 mM MgCl_2_ or 5 mM Lanthanoids) were inferred from ^1^H,^13^C HMQC spectra using samples containing 80–110 µM concentration of MIL^proS^V^proS^AT LmUGP. *K*_Ds_ for the coordination of divalent and trivalent metals to UTP were obtained from series of ^1^H NMR spectra at metal concentrations ranging from 0 to 1400 µM. UTP concentration was 239.2 µM for the titration of MgCl_2_ and LaCl_3_, and 250 µM for the titration of LuCl_3_, EuCl_3_ and CeCl_3_.

Samples for the measurement of PREs contained 199 µM MIL^proS^V^proS^AT LmUGP, 1.5 mM UTP and 55 µM of either MgCl_2_ or MnCl_2_. PCS were measured from samples containing 100 µM MIL^proS^V^proS^AT LmUGP, 3.5 mM UTP and 2.1 mM of one of the following lanthanoid (LuCl_3_ and LaCl_3_ as diamagnetic references, and TbCl_3_, TmCl_3_, EuCl_3_ and CeCl_3_ as paramagnetic samples). In all the cases, ligands and metals were dissolved at high concentrations in the aforementioned buffers and the pH* was carefully adjusted to minimize dilution effects and pH-artifacts. LuCl_3_, LaCl_3_, TbCl_3_, TmCl_3_ and EuCl_3_ were obtained from Sigma-Aldrich. CeCl_3_ was purchased from Alfa Aesar, whilst MgCl_2_ and MnCl_2_ were acquired from Merck.

### NMR experiments

All NMR samples were prepared in 3 mm NMR tubes at a final volume of 0.16–0.17 mL. NMR experiments were conducted at 293 K. Spectra were processed with *Topspin 4.0.6* (Bruker) and analyzed using *CCPNMR Analysis 2.4.2* software suit (Vranken et al. 2005). ^1^H chemical shifts were referenced to the DSS-d_6_ peak, and ^13^C signals were referenced indirectly. Unless otherwise stated, NMR experiments were acquired on a 500 MHz Bruker or on a 600 MHz Avance III spectrometers equipped with TCI cryogenic probes. 2D ^1^H,^13^C HMQC spectra^5^ were acquired with 137 ms acquisition time and a spectral window of 3.7 or 7.5 ppm in the direct dimension. In the indirect dimension, the spectral window was set to 18 or 19 ppm with 1024, 512 or 256 increments. The relaxation delay was set to 1.5 s and 4 to 32 transients were acquired. Data was apodized with a QSINE window function, FIDs were zero-filled and forward linear-predicted (16 LP coefficients) prior Fourier-transformation, affording a 2048 × 2048 data matrix with a spectral resolution of 0.63 Hz and 1.16 Hz in the direct and indirect dimensions, respectively. For more details see Table S3.

Residue type identification was achieved using the following samples: [*U*-^15^N,^2^H], ε-[^13^C,^1^H_3_]-Met-labeled (M), [*U*-^15^N,^2^H], δ1-[^13^C^1^H_3_]- Ile-labeled (I), [*U*-^15^N,^2^H], δ2-[^13^C,^1^H^3^]-Leu, γ2-[^13^C^1^H_3_]-Val-labeled (L^proS^V^proS^), [*U*-^15^N,^2^H], γ2-[^13^C^1^H_3_]-Val-labeled (V^proS^), [*U*-^15^N,^2^H], β-[^13^C^1^H_3_]-Ala-labeled (A) and [*U*-^15^N,^2^H], ε-[^13^C,^1^H_3_]-Met, δ1-[^13^C^1^H_3_]-Ile, δ2-[^13^C,^1^H^3^]-Leu, γ2-[^13^C^1^H_3_]-Val, β-[^13^C^1^H_3_]-Ala, γ-[^13^C^1^H_3_]-Thr-labeled (MIL^proS^V^proS^AT) LmUGP. Samples contained 5 mM MgCl_2_ and protein concentrations ranging from 250 to 450 µM.

4D HMQC-NOESY-HMQC experiments (Tugarinov et al. 2005) of the apo and UDP-Glc bound conformations of LmUGP were acquired with a sample containing 450 µM MIL^proS^V^proS^AT methyl-labeled LmUGP and 5 mM MgCl_2_ before and after the addition of 12 mM UDP-Glc, respectively. Both experiments were measured at the Utrecht NMR SONNMRLSF facility on a 900 MHz or on a 950 MHz Bruker NMR machine equipped with a cryoprobe, respectively. For both acquisitions the mixing time was set to 180 ms. Apo conformation was measured using 30% non-uniform sampling (Robson et al. 2019) (NUS) according to a Poisson Gap sampling schedule (Hyberts et al. 2010) with 11,466 complex NUS data points in a grid of 70(^13^C) × 84(^1^H) × 52(^13^C) points in the indirect dimensions. The UDP-Glc bound conformation was acquired with 31.36% NUS Poisson Gap sampling schedule with 10,764 complex NUS data points in a grid of 66(^13^C) × 80(^1^H) × 52(^13^C) points in the indirect dimensions. For both experiments 512 points were acquired in the direct dimension, with 4 transients and a recovery delay of 1 s. Spectra were processed on a Mac-BookPro running Yosemite 10.10.5 using recursive Multi-Dimensional Decomposition (MMD, Bruker).

Transverse ^1^H_M_-Γ_2_ PRE relaxation rates were measured at 600 MHz using a 2D ^1^H,^13^C HMQC-based pulse scheme described elsewhere (Venditti et al. 2011). Seven relaxation delays (t = 0, 6, 15, 25, 36, 50 and 100 ms) were acquired in an interleaved manner with 102 ms acquisition time and spectral window of 4.2 ppm in the direct dimension. In the indirect dimension, the spectral window was set to 19 ppm with 512 increments. The recovery delay was 1.5 s, and 4 scans were acquired per experiment. Measurements were repeated twice. Data was apodized with a QSINE window function, and FIDs were zero-filled prior to Fourier-transformation to give a 2048 × 2048 data matrix. Spectra were manually phased, and decays in cross-peak intensity were fitted to an exponential decay model (Iwahara et al. 2007) using an in-house *Matlab R2019b* script. Only peaks showing an intensity ≥ 3 σ of spectral noise-floor at t = 6 ms were selected for fitting. Experimental random errors in *R*_2_ rates were estimated as one standard deviation from a Monte Carlo simulation (Kamath and Shriver 1989) with 1,000 iterations of single exponential fits with the spectral noise-floor taken as an estimate of random uncertainties in peak intensities. Experimental Γ_2_ were calculated as the difference in transverse relaxation rates between the sample containing MnCl_2_ (*R*_2,para_) and the sample loaded with MgCl_2_ (*R*_2,dia_) according to Eq. (1):

1 $$\Gamma_{2} = R_{2,para} - R_{2,dia}$$

PCS were measured at 500 MHz from 2D ^1^H,^13^C HMQC spectra as explained above using a spectral window of 3.7 × 19 ppm and 1024 × 256 increments in the direct and indirect dimensions, respectively. The relaxation delay was set to 1.5 s and 4 transients were acquired. Experimental PCS were calculated in Hz as the difference in chemical shifts in the proton dimension between the diamagnetic and the paramagnetic sample according to Eq. (2):

2 $$\delta^{PCS} = \delta \left( {{}_{{}}^{1} H} \right)_{para} - \delta \left( {{}_{{}}^{1} H} \right)_{dia}$$

Rotational correlation times $\tau_{r}$ of [*U*-^15^ N,^2^H] labeled LmUGP at 50 to 335 µM protein concentrations have been estimated using TRACT experiments (Lee et al. 2006) at 500 and 600 MHz. The relaxation delay was set to 2 s. Experiments were measured for 16 transients with 25 increasing delays of up to 0.4 s. Data were integrated from 8–10 ppm, normalized and fitted to an exponential decay model for determination of average ^15^ N *R*_α_ and *R*_β._ Samples contained 5 mM MgCl_2_.

### Calculation of Γ_2_ PREs

The contribution to PRE from Curie spin relaxation of Mn^2+^ at 600 MHz can be neglected, since it accounts only for 0.14% on the total relaxation enhancement in ^1^H. Therefore, in this case PRE rate arising from the dipole–dipole interaction between a nucleus and an unpaired electrons (Γ_2_) is described by the Solomon-Bloembergen-Morgan (SBM) Eq. (3) (Bloembergen and Morgan 1961).

3 $${\Gamma }_{2} = \frac{1}{15}\left( {\frac{{\mu_{0} }}{4\pi }} \right)^{2} \frac{{\gamma_{I}^{2} g^{2} \mu_{B}^{2} S\left( {S + 1} \right)}}{{r^{6} }}\left( {4\tau_{c} + \frac{{13\tau_{c} }}{{1 + \omega_{S}^{2} \tau_{c}^{2} }} + \frac{{3\tau_{c} }}{{1 + \omega_{I}^{2} \tau_{c}^{2} }}} \right)$$

where *r* is the distance between the paramagnetic centre and the observed nucleus; $\mu_{0}$ is the permeability of vacuum; $\gamma_{I}$, the nuclear gyromagnetic ratio; *g,* the electron *g*-factor; $\mu_{B}$, the electron Bohr magneton; *S*, the electron spin quantum number; $\omega_{I}$ and $\omega_{S}$, the proton and electron Larmor frequencies, respectively; $\tau_{c}$, the PRE correlation time defined as $\tau_{c}^{ - 1} = \tau_{r}^{ - 1} + \tau_{s}^{ - 1}$; with $\tau_{s}$, the electron relaxation time, which has been reported to be 9.6 ns for Mn^2+^ at 600 MHz (Iwahara et al. 2007). Calculated Γ_2_ were multiplied by 0.0241 to be comparable with the measured values, as explained in Results and Discussion. No crystal structure is available for LmUGP in complex with UTP and a metal ion. Therefore, we used the following crystal structures of LmUGP as stand alone or as an ensemble: Apo (pdb 2OEF), UDP-Glc (pdb 4M2A), and in complex with UTP analog dUpCpp (pdb 4M28). Coordinates of the metal position were fitted via a non-linear gradient descent using the Broyden-Fletcher-Goldfarb-Shanno (BFGS) algorithm (Fletcher 1988) for non-linear least-square minimisation of the cost function described in Eq. (4) (Orton et al. 2020).

4 $$cost_{ensemble} = \mathop \sum \limits_{i} \frac{{\left[ {\mathop \sum \nolimits_{m} ({\Gamma }_{2,i}^{obs} - {\Gamma }_{2,m,i}^{calc} )} \right]^{2} }}{{S_{{{\Gamma }_{2,i} }}^{2} }}$$

where ${\Gamma }_{2}^{obs}$ and ${\Gamma }_{2}^{calc}$ are the observed and calculated PREs, respectively. Index *m* is for atoms that are common between models, index *i* runs over every atom in the structure and $S_{{{\Gamma }_{2,i} }}$ corresponds to the experimental uncertainty in the Γ_2_ of spin *i*. Error in the fitting of the metal position corresponds to one standard deviation.

### Fitting of alignment tensors from PCS data

Anisotropic magnetic susceptibility tensors $\Delta \chi$ induced by lanthanoid ions were obtained by fitting of experimental PCS to Eq. (5) as described in *Paramagpy* software package (Orton et al. 2020).

5 $$\delta^{PCS} = \frac{1}{{4\pi r^{5} }}\left[ {\begin{array}{*{20}c} {x^{2} - z^{2} ,} & {y^{2} - z^{2} ,} & {\begin{array}{*{20}c} {2xy,} & {2xz,} & {2yz} \\ \end{array} } \\ \end{array} } \right] \cdot \left[ {\begin{array}{*{20}c} {\Delta \chi_{xx} } \\ {\Delta \chi_{yy} } \\ {\begin{array}{*{20}c} {\Delta \chi_{xy} } \\ {\Delta \chi_{xz} } \\ {\Delta \chi_{yz} } \\ \end{array} } \\ \end{array} } \right]$$

Being *x*, *y*, *z* the coordinates of the metal centre and $\Delta \chi_{xx} ,\;\Delta \chi_{yy} , \;\Delta \chi_{xy} ,\;\Delta \chi_{xz} , \;\Delta \chi_{yz}$ five explicit parameters that characterise the $\Delta \chi$ tensor. Same combinations of structural models as described in the calculation of Γ_2_ were used here. Fittings included corrections for residual dipolar anisotropic shifts (RADS) and for residual anisotropic chemical shifts (RACS), as indicated in *Paramagpy*. The latter was achieved using standard chemical shift anisotropy (CSA) tensors for ^13^C spins (Cornilescu and Bax 2000). Quality of fitted $\Delta \chi$ tensors was evaluated via a bootstrapping approach with 1,000 iterations and 80% randomly sampled data at each specific iteration. Errors are reported as one standard deviation.

### Calculation of Q factors

Goodness of fitting between observed and calculated PREs and PCS was evaluated by calculating Q factors according to Eq. (6)

6 $$Q_{ensemble} = \sqrt {\frac{{\mathop \sum \nolimits_{i} \left[ {\left( {\mathop \sum \nolimits_{m} \left[ {{\text{a}}_{i}^{obs} - {\text{a}}_{m,i}^{calc} } \right]} \right)^{2} } \right]}}{{\mathop \sum \nolimits_{i} \left[ {\left( {\mathop \sum \nolimits_{m} \left[ {{\text{a}}_{i}^{obs} } \right]} \right)^{2} } \right]}}}$$

where index *m* is for atoms that are common between models, index *i* runs over every atom in the structure and *a* represents either ${\Gamma }_{2}$ or PCS.

### Use of chemical shift data in MAP-XSII

*MAP-XSII* requires chemical shift data to compute assignments, although it can easily lead to assignment errors (Pritisanac et al. 2017). To overcome this problem, chemical shifts were predicted using CH3Shift (Sahakyan et al. 2011) and SHIFTX2 (Han et al. 2011) and weighted the by a factor of 10^–12^, de facto excluding them from the calculations. The content of the remaining input files is explained under Results and Discussion.

### Calculation of dissociation constants

Titrations were used to derive Euclidean chemical shift perturbances (CSPs) $\Delta \nu_{eucl}$ according to Eq. (7).

7 $$\Delta \nu_{eucl} = \sqrt {\Delta \nu_{H}^{2} + \Delta \nu_{C}^{2} }$$

$\Delta \nu_{H}$ and $\Delta \nu_{C}$ are CSPs in the respective dimensions in units of Hz. In a simple two states model (Eq. 8), observed $\Delta \nu_{obs}$ at a given total ligand concentration *L*_*t*_ are linked to the dissociation constant *K*_D_ via the law of mass action (Williamson 2013) (Eq. 9).

8 $$P + L \rightleftharpoons PL$$

9 $$\Delta \nu_{obs} = \frac{{\left( {P_{t} + L_{t} + K_{D} } \right) - \sqrt {\left( {P_{t} + L_{t} + K_{D} } \right)^{2} - 4P_{t} L_{t} } }}{{2P_{t} }}\Delta \nu_{\max }$$

where *P*_*t*_ is the total protein concentration, and $\Delta \nu_{\max }$ is the maximum CSP at ligand saturation for each signal. Titration curves for nonlinear least-squares global fitting were selected according to the magnitude of the CSPs at the highest ligand concentration: CSPs larger than the mean of all CSPs + two standard deviations were used to derive a *K*_*D*_ value. Global fittings were performed using in-house *Matlab* scripts. Errors were determined from a Monte Carlo approach with 1,000 iterations as previously described (Arai et al. 2012), and are given as one standard deviation.

## Results and discussion

**Fig. 2:**
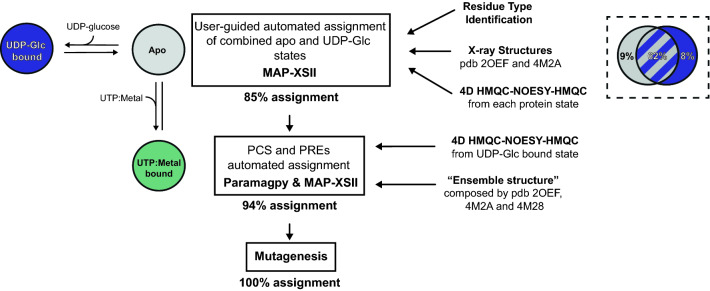
Overall strategy for the structure-based assignment of MIL^proS^V^proS^AT LmUGP using multiple protein states. Central to this strategy was the acquisition of 4D methyl-methyl NOESY experiments, which delivered information about neighbouring methyl groups in the apo and UDP-Glc bound protein states (grey and blue, respectively). In a first step, data from each protein state was independently loaded into *MAP-XSII.* Comparison of independent assignments from each state revealed a 62% overlapping. As expected, the apo state delivered NOE cross-peaks not observed in the UDP-Glc bound state, and vice versa*.* Analysis of such extra NOE peaks allowed ca. 8% extra assignments exclusively for each state, as shown in the upper right insert. Inclusion of the extra assignments from the complementary state as fixed in *MAP-XSII* calculations enlarged the assignment to a total 85%. PCSs of the 85% assigned residues were then used to derive initial, approximate Δ$\chi$ tensors for each lanthanide ion with *Paramagpy.* Addition of experimental and calculated PCSs from all observed residues into *MAP-XSII* expanded the assignment to 93% resonances. PREs allowed the assignment of only 2 methyl resonances (94%), and were used as a validation tool. Finally, directed mutagenesis of the remaining 12 unassigned amino acids delivered the assignment of all MIL^proS^V^proS^AT methyl-group resonances

The overall workflow can be divided into three sequential steps, as seen in Fig. 2. First, methyl-methyl NOEs were measured from the “apo” (pdb 2OEF) and “UDP-Glc” bound (pdb 4M2A) LmUGP states. These two states exhibit the largest RMSD (3.4 Å) among all available crystal structures, and were therefore selected to maximize differences between the two sets of NOE restraints. Methyl walking delivered a 85% preliminary assignment of methyl groups, which was used as starting point for refinement of the assignment based on paramagnetic NMR. Apo LmUGP does not specifically interact with di- and trivalent ions in solution. However, metal ions can occupy the enzyme binding pocket when in complex with UTP, as it is shown for Mg^2+^ as a representative example in Figure S1. PCS and PREs were measured using samples at specific UTP and metal ratios. Experiments with paramagnetic metals expanded the assignment to a total 94%, confirming the assignments allocated from methyl-methyl NOE experiments. Finally, unassigned methyl groups were mutated to complete the assignment.

### Identification of residue types

**Fig. 3:**
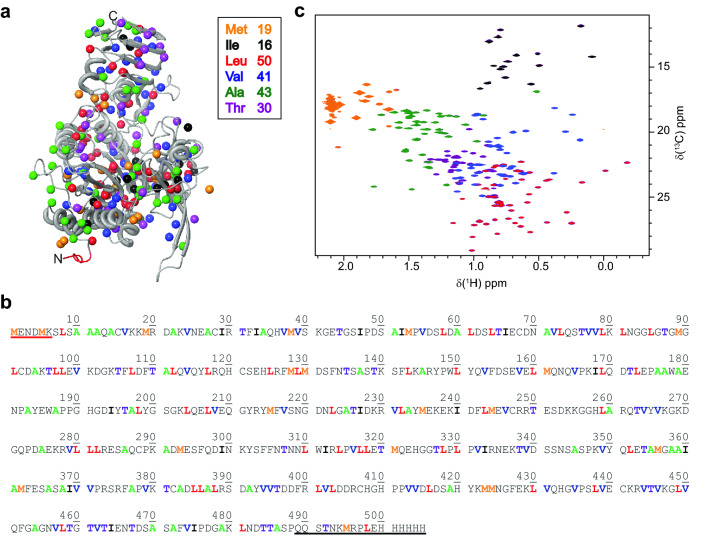
Distribution of methyl groups in LmUGP and residue type identification. **a** Crystal structure of LmUGP in the apo state (pdb 2OEF). The carbons of the methyl groups are highlighted as spheres. Only the *pro-*(*S*) methyl groups of Leu and Val residues are shown. A summary of amino acids labeled and the colour scheme used is provided in the insert. **b** Amino acid sequence of LmUGP showing the distribution of methyl-labeled amino acids. Colour code like in (a). **c** Superposition of ^1^H,^13^C HMQC spectra of apo LmUGP. Each spectrum is coloured individually (M orange, I black, L^proS^V^proS^ red, V^proS^ blue, A green and MIL^proS^V^proS^AT violet). The order of the spectra in the superimposition was selected to match the colour scheme in **a**, **b**. Modelled *N*-terminus is indicated in red in **a** and **b**, and *C*-terminus with low electron density is highlighted in black in **b**. All samples were measured at 293 K and 500 MHz with varying protein concentrations ranging between 200 and 450 µM

The first step in any structure-based assignment is the correlation of the methyl group resonances with their respective amino acid types. Here, Ala-β, Ile-γ1, Leu-δ2, Val-γ2, Met-ε and Thr-γ methyl groups were [^13^C,^1^H_3_]-methyl labeled, yielding a total of 199 [^13^C,^1^H_3_]-methyl groups to be assigned. The labeling scheme was chosen because it combines maximal methyl probe density with minimal signal crowding. As can be seen in Fig. 3a, b, labeled methyl groups are evenly distributed over the whole enzyme. Analysis of peak intensities from a single ^1^H-^13^C HMQC spectrum suggested a uniform incorporation of isotopes into all six amino acid types (Fig. S2).

To identify the amino acid types we prepared six methyl labeled samples, according to the following labeling schemes: M-, I-, L^proS^V^proS^-, V^proS^-, A- and MIL^proS^V^proS^AT-methyl labeled LmUGP. Assignment of Ala-, Ile-, Met- and Thr-methyl groups was straightforward from a simple comparison between spectra. Discrimination between Leu- and Val-methyl resonances was achieved based on leucine selective unlabeling (Mas et al. 2013). For the apo state we found 196 out of 199 expected resonances distributed as follows: 43/43 alanine, 16/16 isoleucine, 48/50 leucine, 19/19 methionine, 30/30 threonine and 40/41 valine resonances (Fig. 3c). Addition of saturating concentrations of UDP-Glc resolved signal overlapping, allowing the observation of 199 out of 199 expected resonances.

### Preliminary assignment of methyl groups based on 4D HMQC-NOESY-HMQC experiments

**Fig. 4:**
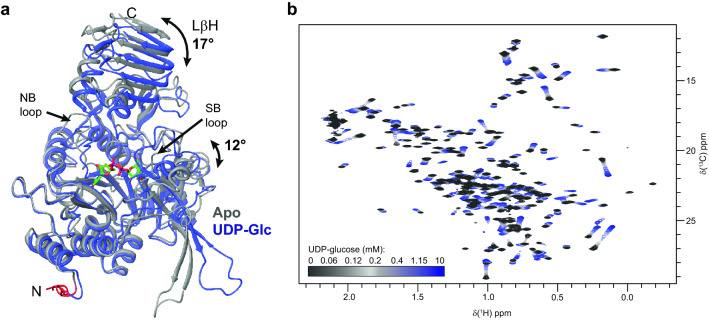
Saturation of LmUGP with UDP-glucose induces conformational changes, allowing two sets of short-range structural restraints in non-identical chemical environments. **a** Superimposition of crystal structures of LmUGP in the apo state (grey) and bound to UDP-Glc (UDP-Glc state, blue), pdb codes 2OEF and 4M2A, respectively. UDP-Glc is shown in sticks representation, and modelled residues are indicated as a red cartoon. Upon UDP-Glc binding, the left-handed parallel β-sheet (LβH) constituted by residues 391–488 tilts forward by 17°, and the sugar-binding (SB) loop and adjacent residues turn by 12° toward the sugar moiety. Additionally, the nucleotide-binding (NB) loop experiences a significant spatial reorientation. **b** Superimposition of ^1^H,^13^C HMQC spectra corresponding to the titration of a 154 µM concentration MIL^proS^V^proS^AT labeled LmUGP with UDP-Glc. Spectra were acquired at 500 MHz and at 293 K

Crystallographic studies have shown that LmUGP undergoes a large conformational transition upon binding of UDP-Glc (Steiner et al. 2007) According to available crystal structures (pdbs 2OEF and 4M2A), conformational changes involve rearrangement of the *C*-terminal domain together with functional loops of the catalytic domain, forcing the enzyme to adopt a more “compacted” shape (Fig. 4a). The structural rearrangement is also apparent in ^1^H,^13^C HMQC spectra, where a titration of MIL^proS^V^proS^AT LmUGP with UDP-Glc produced observable CSPs for virtually every methyl group (Fig. 4b). Based on these structural differences, it can be postulated that 4D HMQC-NOESY-HMQC experiments acquired from the apo and UDP-Glc bound protein states may deliver a subset of unique methyl-methyl NOEs. Such unique structural restraints can be exploited to expand the completeness of the methyl assignments beyond the limits of a single protein conformation. Assignments can be easily transferred between apo and UDP-Glc bound states from simple ligand titrations, since both protein states interconvert in fast or close-to-intermedium exchange in the NMR time scale.

**Fig. 5:**
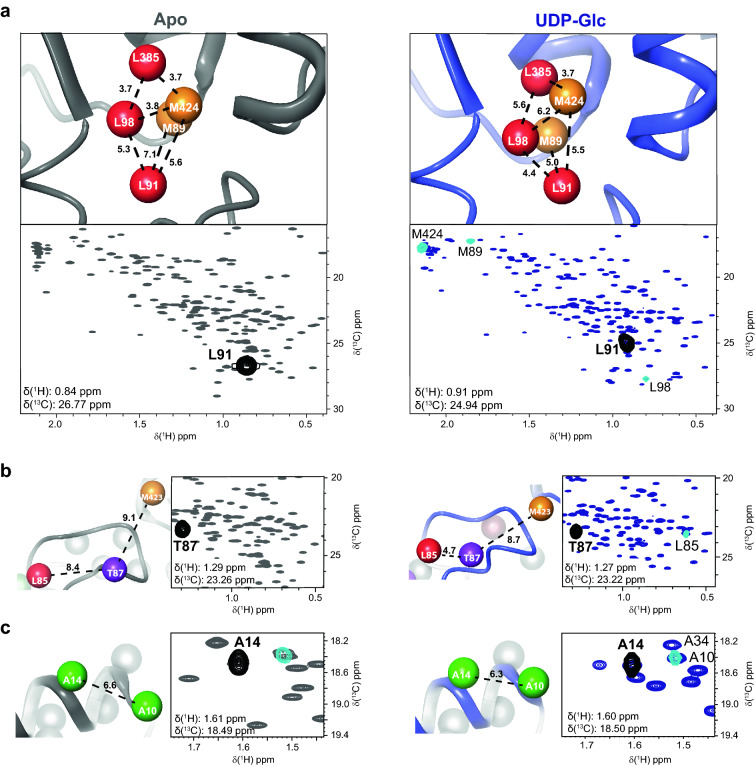
Structural changes upon ligand binding facilitate assignments. **a** Example of a methyl–methyl NOE cluster of spatially neighbouring amino acids. Top panels: Crystal structures of LmUGP in the apo and in the UDP-Glc states (grey and blue, pdb 2OEF and 4M2A, respectively) with the methyl carbons represented as spheres. Bottom panels: Superposition of F1(^13^C)/F2(^1^H) planes of the 4D HMQC-NOESY-HMQC experiments (black is the auto peak, turquoise are NOE cross-peaks) and of the ^1^H,^13^C HMQC spectra (grey or blue for apo or UDP-Glc states, respectively) recorded with 450 µM of MIL^proS^V^proS^AT LmUGP in the absence (left) or presence (right) of 12 mM UDP-Glc. The F3(^13^C)/F4(^1^H) frequencies for plane selection are given on the bottom left of each panel. **b** Methyl groups of L85 and T87 come close in space upon ligand binding enabling the assignment of L85 exclusively in the UDP-Glc bound state. **c** Changes in the environment around A10 and A34 upon UDP-Glc binding resolves signal overlapping, allowing the assignment of A14. All distances are given in Å. Spectra were acquired at 900 MHz (apo state) or 950 MHz (UDP-Glc state) at 293 K

4D HMQC-NOESY-HMQC experiments provided short-range structural information through NOE connectivities between neighbouring methyl groups. Methyl-methyl NOEs were analyzed from F1(^13^C)/F2(^1^H) planes as previously described (Flugge and Peters 2018). NOE patterns showing mutual connectivities were grouped into clusters allowing univocal assignments of methyl groups based on distance information from the crystal structure, in a process usually described as “methyl walk”. A comparative example of such “methyl walk” for the same region of the apo and UDP-Glc states of LmUGP can be found in Fig. 5a. As expected, each protein state shows a different pattern of NOE correlations in accordance to the distances observed in the crystal structures. Note that some amino acids could only be assigned in a specific protein state (*e.g.* Leu91 after saturation with UDP-Glc).

4D NOESY experiments afforded a total of 486 and 531 methyl-methyl NOEs in the absence and presence of UDP-Glc, respectively. From these NOE cross peaks, 189 NOE connections were only found in the apo state, whereas 234 NOE connections were unique to the UDP-Glc state. The higher number of methyl-methyl NOEs in the bound state as compared to the apo correlates with a more compacted, closed enzyme structure, as observed in the crystal structures. It is worth noting that differences in relaxation rates between protein states associated to local protein dynamics or the existence of invisible states could also contribute to the observed differences. Two main mechanisms contributed to the unique NOE cross-peaks observed in each protein state. Firstly, structural reorientations of specific protein motifs alter the distance between methyl groups, allowing the observation of new NOE cross-peaks. A good example is L85, whose methyl moiety approaches T87 upon UDP-Glc addition permitting its unequivocal assignment (Fig. 5b). Secondly, changes in chemical environment in methyl groups after UDP-Glc addition allowed the resolution of overlapping signals. For example, resonances from A10 and A14 superimpose in the apo state. Addition of UDP-Glc does not alter the distance between methyl groups. However, changes in the chemical environment due to the presence of the ligand in its binding pocket allowed the discrimination between methyl signals (Fig. 5c).

Next, we used *MAP-XSII* (Xu and Matthews 2013) as an automated method to obtain NOE-based preliminary assignments of each enzyme state. We selected *MAP-XSII* because it allows the simultaneous use of short- and long-distance spatial restraints (NOEs and paramagnetic NMR, respectively). It also has a good assignment reliability and requires short computation times (Pritišanac et al. 2020). It is worth mentioning that other algorithms allowing simultaneous use of multiple restraints like *PRE-ASSIGN* (Venditti et al. 2011) and *FLAMEnGO2.0* (Chao et al. 2014) could also be used, although were not systematically explored in this study. *MAP-XSII* uses multiple parallel repetitions based on a Metropolis Monte Carlo (MMC) swapping routine to perform automated assignments according to the experimental restraints supplied. In a first round, methyl resonances from each enzymatic state were assigned independently. Therefore, NOE connectivities and structural models (pdb entries 2OEF for apo and 4M2A from UDP-Glc states) corresponding to each protein state together with amino acid residue types were used as inputs for the assignment in two separated runs. It should be mentioned that all available LmUGP crystal structures show two regions with poor electron density. These regions comprise the first seven *N*-terminal residues including M1 and M5, and the last 17 amino acids including T492, M495 and L498 (Fig. 3c). The web server *ModLoop* (Fiser and Sali 2003) was used to model structural gaps, delivering a consistent orientation for the *N*-terminus in ten iterative runs. The *C*-terminus was excluded from the final models because *ModLoop* produced ambiguous orientations, probably due to weak amino acid interactions.

For the initial assignment of apo and UDP-Glc bound states, 20 MMC trials were performed for each state with cut-off distances spanning between 5 and 11 Å. Each structural model together with its corresponding spatial NOE-based restraints were separately computed. Cut-offs were selected to maximize the number of signals consistently assigned to the same residue in 20 MMC runs, and corresponded to 10 and 7 Å for the apo and UDP-Glc bound states, respectively (see Fig. S3). From a total of 199 methyl groups to be assigned, 141 and 139 were consistently assigned to the same residue in the apo and UDP-Glc states, correspondingly. Notably, only 123 assignments (62% total assignment) were shared between both protein states.

As previously explained, useful spatial information is also encoded in the NOE signals producing unique assignments for each protein state. Such NOE cross-peaks can be considered as key signals, which act as bridges between otherwise isolated NOE clusters or methyl groups in each protein state. To exploit this information, we repeated the automated assignment using *MAP-XSII* although this time with an important caveat: assignments solely obtained in one state were included as fixed assignments during the calculations of the complementary state. Runs for each protein state produced almost identical results, expanding the assignment to a total 85% of the methyl groups (169 assignments). Subsequent manual methyl-walks of both NOE datasets confirmed the assignments obtained by *MAP-XSII*.

### Lanthanide-induced PCS allow expansion of the assignment

**Fig. 6:**
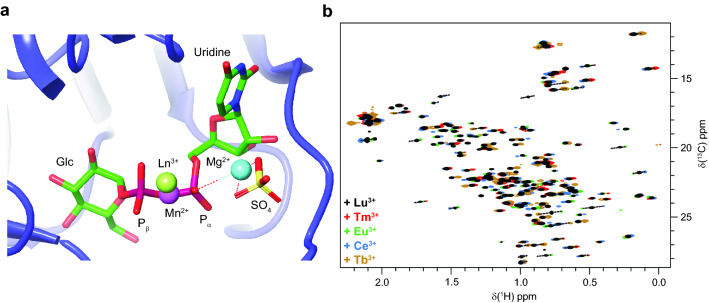
Metal binding sites and PCSs. **a** Crystal structure showing the location of Mg^2+^ (cyan) in the presence of UDP-Glc (pdb 4M2A). The pyrophosphate group from UDP-Glc and one molecule of SO_4_ coordinate the metal ion. Free pyrophosphate (PP_i_) was substituted by SO_4_ during the crystallization to approximate the metal position of the PPi:Mg^2+^ release step after the enzymatic reaction. Coordinates from combined fit of Tb^3+^, Tm^3+^ and Eu^3+^ (Ln^3+^ green), and Mn^2+^ (violet) obtained in the presence of UTP are shown as comparison. **b** Superimposition of ^1^H,^13^C HMQC spectra acquired for a sample of MIL^proS^V^proS^AT labeled LmUGP in the presence of UTP and 50% metal binding site occupation (Lu^3+^ black, Tm^3+^ red, Eu^3+^ green, Ce^3+^ blue, Tb^3+^ yellow). Spectra were acquired at 500 MHz and 293 K

No crystal structure of LmUGP in complex with UTP and Mg^2+^ is currently available. However, ab initio quantum mechanics/molecular mechanics (QM/MM) calculations have indicated that LmUGP coordinates the complex UTP:Mg^2+^ in the first step of the catalytic cycle (Führing et al. 2013). After the enzymatic reaction, Mg^2+^ is weakly hold in the binding pocket by coordination to UDP-Glc and PPi, being eventually released as a PPi:metal complex (Fig. 6a). Here, we took advantage of this feature and substituted Mg^2+^ with lanthanide ions in the presence of UTP. La^3+^ was used as diamagnetic reference for Ce^3+^ and Eu^3+^, whilst Lu^3+^ served as diamagnetic control for Tb^3+^ and Tm^3+^, in accordance with their ionic radii (D'Angelo et al. 2011). During binding, the metal ion coordinates to UTP. Due to its central location, paramagnetic relaxation enhancements (PREs) impeded the observation of a significant number of residues around the metal ion. This disadvantage could be partially compensated with the use of Ce^3+^ and Eu^3+^. Such lanthanides induce small paramagnetic effects, allowing sampling of methyl residues located close to the lanthanide ions. The use of Tb^3+^ and Tm^3+^ delivered significant PCS as far as 40 Å from the metal position. Magnetic anisotropy Δχ tensors were calculated from independent or shared metal centres for all lanthanide ions, affording in all the cases almost identical metal centre positions. PCS in the ^13^C dimension are prone to errors due to their lower resolution and to their larger residual chemical shift anisotropy when compared to protons. Consequently, only shifts in the ^1^H dimension were used for Δχ tensor refinement.

Mg^2+^ coordinates oxygens from α-and β-phosphates from UTP, establishing no further interactions with neighbouring amino acids (Führing et al. 2013). In other words, Mg^2+^ only occupies the protein metal centre as a UTP:metal complex. It was therefore crucial to ensure protein saturation with UTP. With protein concentrations of about 100 µM, this was achieved with a 35-fold ligand excess. For LmUGP, the equilibrium between free, UTP- and UTP:metal-bound protein species is in fast exchange in the NMR timescale, as it is shown for Mg^2+^ as a representative example in Fig S4. In such a case, it is possible to extract PCS and PREs when the fraction of UTP:metal:LmUGP complex matches between paramagnetic metals and their diamagnetic references. In addition, undesired paramagnetic effects could be observed in solvent-exposed methyl groups at free metal ion concentrations over ~ 80 µM. As a consequence, one cannot simply saturate the protein with UTP:metal complex, because a large fraction of free metal will always be present inducing unspecific paramagnetic effects. In a nutshell, accurate PCS and PREs can only be obtained when the exact fraction of UTP:metal:LmUGP (UMP) complex is known, and when the free lanthanide concentration is kept well below ~ 80 µM.

**Fig. 7:**
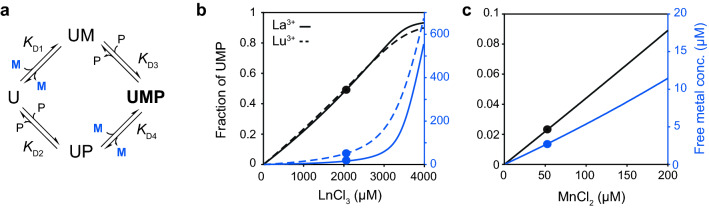
A four-states binding model allows to predict the fraction of free and protein-bound metal. **a** Binding model describing the interaction of UTP (U) with a metal ion (M) and LmUGP (P). UM, UP and UMP denote UTP:metal, UTP:LmUGP and UTP:metal:LmUGP complexes, respectively. Specific PCS and PREs are observed only from the complex UMP (highlighted in bold), whilst free metal concentration (blue) needs to be kept as low as possible in order to minimise unspecific paramagnetic effects. **b**, **c** Fraction of UMP complex (black) and free metal concentration (blue) as a function of total metal concentration. **b** Corresponds to LaCl_3_ and LuCl_3_ as representative ions for lanthanides (solid and dashed lines, respectively), and **c** relates to MnCl_2_. Dots indicate the conditions used in this study. Plots were created from experimental data described in Table 1 using Eqs. (3) and (4) from supplementary information

**Fig. 8:**
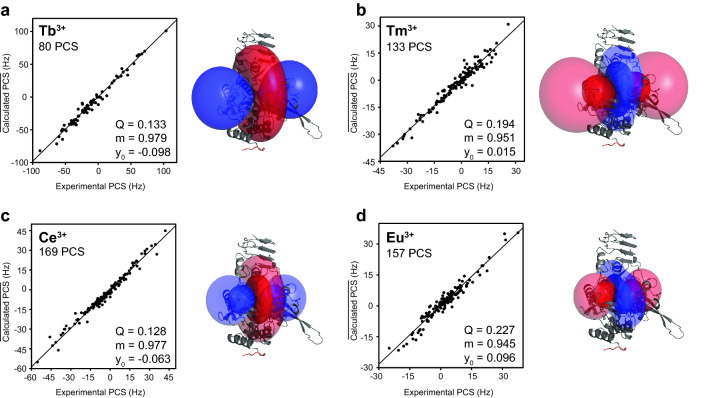
Correlations of experimental *vs.* calculated PCS and anisotropic magnetic susceptibility tensors for **a** Tb^3+^, **b** Tm^3+^, **c** Ce^3+^ and **d** Eu^3+^. Experimentally determined and calculated PCS values are plotted in the x- and y-axis, respectively (in Hz). Calculated PCS were obtained as an average from all structures in the ensemble. The quality of the $\Delta \chi$ tensors was evaluated according to the three parameters displayed as inserts in each correlation plot: Q factors (Eq. 6), slopes (m) and y-axis intercepts (y_0_). The slope of the plot should be close to one and intercepts with the y-axis should be close to zero. The metal position was not fixed in the calculation of the Δχ tensor. Each panel shows the crystal structure of LmUGP in UDP-Glc state (pdb 4M2A) in cartoon representation with PCS isosurfaces contoured at ± 50 Hz and ± 10 Hz (opaque and transparent color, respectively). Note that for Tb^3+^ only isosurfaces at ± 50 Hz have been plotted. Blue and red isosurfaces represent regions with positive and negative PCSs, respectively. Modelled *N*-terminus is indicated as a red cartoon

To obtain a precise control over the experimental set-up we developed a four-states binding model describing the interaction between UTP, a single metal ion and LmUGP (Fig. 7a). Dissociation constants *K*_D1-3_ were experimentally inferred or approximated from simple titrations as explained in supporting information. Binding isotherms for the calculation of *K*_D1_ can be inspected in Fig. S5. Fig S6 shows the binding isotherms corresponding to *K*_D2_ and *K*_D3_ for the binding of UTP to MIL^proS^V^proS^AT LmUGP in the presence and absence of MgCl_2_ as a representative example. As a quality control, we compared the *K*_D1_ for the complexation of Mg^2+^ to UTP obtained from NMR titrations with a previously reported value from isothermal titration calorimetry (Zea et al. 2008). The outcome from both methods was almost identical, reassuring the accuracy of our results. With this data in hand, only four parameters needed to be calculated: concentration of UTP:metal (UM), UTP:LmUGP (UP), UTP:metal:LmUGP (UMP) complexes and *K*_D4_. They were approximated via nonlinear least-squares minimization of the system of Eq. (3) described in supporting information. Results are summarized in Table 1. This knowledge allowed us to prepare samples containing virtually identical fractions of UTP:metal bound to LmUGP for every lanthanoid (Fig. 8b). This set-up produced relatively small PCS owing to the low metal binding site occupation selected (50.5% ± 5%). However, it ensured low free metal concentrations (< 60 µM), preventing unspecific paramagnetic effects and enabling the extraction of accurate PCS from [^13^C,^1^H]-HMQC spectra.

**Table 1:** Dissociation constants for the interaction of UTP with di- and trivalent metals (*K*_D1_), LmUGP with UTP in the absence (*K*_D2_) and in the presence of saturation metal concentrations (*K*_D3_)

Metal	*K*_D1_ (µM)	*K*_D2_ (µM)	*K*_D3_ (µM)	*K*_D4_ (µM)
MgCl_2_	82 ± 6 / 70 ± 3^A^		155 ± 4	115 ± 10
LaCl_3_	11 ± 5		152 ± 6	15 ± 7
LuCl_3_	38 ± 17		138 ± 8	46 ± 21
EuCl_3_	28 ± 9	112 ± 2	152 ± 8	38 ± 12
CeCl_3_	13 ± 4		147 ± 3	17 ± 5
TbCl_3_^B^	–		149 ± 12	~ 30^D^
TmCl_3_^B^	–		154 ± 15	~ 30^D^
MnCl_2_^C^	77 ± 12^A^		–	~ 107^D^

*K*_D4_ was approximated as described in the supplementary information

^A^Previously described from isothermal titration calorimetry(Zea et al. 2008)

^B^*K*_D1_ could not be calculated due to line shape broadening induced by strong PREs. *K*_D1_s from lanthanide ions were averaged and used as an approximation in the calculations

^C^*K*_D3_ from Mg^2+^ was used for calculations because high Mn^2+^ concentrations broaden protein signals beyond detection

^D^Approximated values

The lack of a crystal structure of LmUGP in complex with UTP required some special considerations concerning the choice of a proper structural model. When bound to UTP, QM/MM calculations predict a protein arrangement somewhere between the apo (open) and UDP-Glc bound state (post-reactive) (Führing et al. 2013). Therefore, an “ensemble structure” was generated combining the X-ray structures of available protein states populated by the enzyme during the catalytic cycle (see Fig. S7). The ensemble contained the following structures: apo (pdb 2OEF), UDP-Glc bound (pdb 4M2A) and dUpCpp bound states, an analog of UTP (pdb 4M28).

The 169 consistently assigned resonances were used as a starting point for the fitting of magnetic susceptibility tensors with the help of *Paramagpy* (Orton et al. 2020). Signal overlapping and PREs precluded the determination of PCS for some methyl resonances, allowing the extraction of 169 PCS for Ce^3+^, 157 PCS for Eu^3+^, 133 PCS for Tm^3+^ and 80 PCS for Tb^3+^ (Table S4). The “ensemble structure” as well as the individual conformations were subjected to Δχ tensor fitting. The initial paramagnetic centre was predefined to the metal position in the crystal structure with UDP-Glc bound (Fig. 6a, pdb 4M2A), but allowed to move during the fitting. Nevertheless, the unique tensor representations (UTR) showed that in all cases coordinates of the fitted paramagnetic centre converged in a very similar position (< 1.5 Å difference). Fitted metal coordinates in the presence of UTP deviate by almost 4 Å from those observed for the UDP-Glc state. (Fig. 6a and Table S5). This observation is in good agreement with the proposed molecular mechanism for LmUGP, were Mg^2+^ leaves the binding pocket upon synthesis of UDP-Glc in the form of PPi:metal complex (Führing et al. 2013).

Q factors were used for the evaluation of the quality of protein structures determined from PCS, and are listed in Table 2. The use of the “ensemble structure” yielded the lowest Q factors for every lanthanide ion. Our results nicely reflect that LmUGP adopts several conformational states in solution along the catalytic cycle, as defined by crystal structure analysis. Overall, tensors were very robust in the error analysis with the exception of the γ angle of the principal axis of the Ce^3+^ tensor, as summarized in Table S5. PCS from Ce^3+^ were therefore considered as imprecise and excluded from further analysis.

**Table 2:** Q factors derived from measured and calculated PCSs and PREs from combined fits to different structural models

Structural model	Q factor				
	PCSs		PREs		
	Tb^3+^	Tm^3+^	Ce^3+^	Eu^3+^	Mn^2+^
Apo state (pdb 2OEF)	0.158	0.226	0.155	0.246	0.473
UDP-Glc state (pdb 4M2A)	0.159	0.195	0.171	0.226	0.441
In complex with UTP analog dUpCpp (pdb 4M28)	0.181	0.196	0.182	0.265	0.374
“Ensemble structure” (pdb 2OEF, 4M2A, 4M28)	0.133	0.194	0.128	0.227	0.172

$\Delta \chi$ tensors and Γ_2_ were fitted to the apo, UDP-Glc, dUpCpp analog or to an “ensemble structure”

*Paramagpy* provided lists of expected PCSs for all methyl protons, which were used to expand the available assignment. In the next step, these lists together with the experimental PCSs were used as additional input files in a new round of *MAP-XSII*. All PCSs were multiplied by a weighting factor of 10 to match the order of magnitude of PREs normally used in these calculations, as previously described (Flugge and Peters 2018). The NOE list and the crystal structure of the UDP-Glc bound state (pdb 4M2A) were introduced as additional input files. We selected this crystal structure because it showed the closest Q factors to the “ensemble structure” (Table 2). 20 MMC trials were performed for each PCS list with cut-off distances spanning between 7 and 13 Å (Fig. S8). Although the cut-off distances were varied, the results were very similar for every lanthanide ion. Overall, 165 out of 169 assignments previously obtained could be confirmed. The four assignments that could not be reproduced corresponded to methyl groups assigned through NOE correlations which were present only in the apo conformation. *MAP-XSII* generated 22 to 29 new assignments solely based on PCSs, depending on the paramagnetic metal and cut-off distance preferred. However, not every peak was assigned to the same amino acid in every PCS list. As inclusion criteria we selected only amino acids consistently assigned to the same signal in at least two Δχ tensors with opposite orientations (i.e., Tb^3+^ and Tm^3+^ or in Tb^3+^ and Eu^3+^). Following this approach 16 new assignments were obtained, rising the number of assigned methyl groups to 185 (93%). PCSs of all assigned residues were finally used to derive Δχ tensors depicted in Fig. 8.

### Completeness of the assignment using PREs and directed mutagenesis

Free electrons from paramagnetic metals induce fast relaxation of signals from nuclei sited close to the metal centre. The effect is equally pronounced in all directions around the metal ion, allowing for straightforward calculations of metal coordinates. Such calculations are most robust when the paramagnetic metal is uniformly surrounded by methyl groups, as it is the case of LmUGP when bound to the UTP:metal complex. In addition to this, PREs can be used for the verification of the assignments obtained by NOEs and PCS. We selected Mn^2+^ because this metal induces stronger PREs than the lanthanide ions. Furthermore, Mn^2+^ is a bivalent cation having an identical coordination sphere and a similar ionic radius as the natural Mg^2+^ cofactor. MnCl_2_ was added at low concentrations (55 µM) in the presence of UTP, which allowed the measurement of site-specific PREs for methyl groups as close as 10 Å from the metal centre. Reliable PREs could be extracted from 192 signals.

**Fig. 9:**
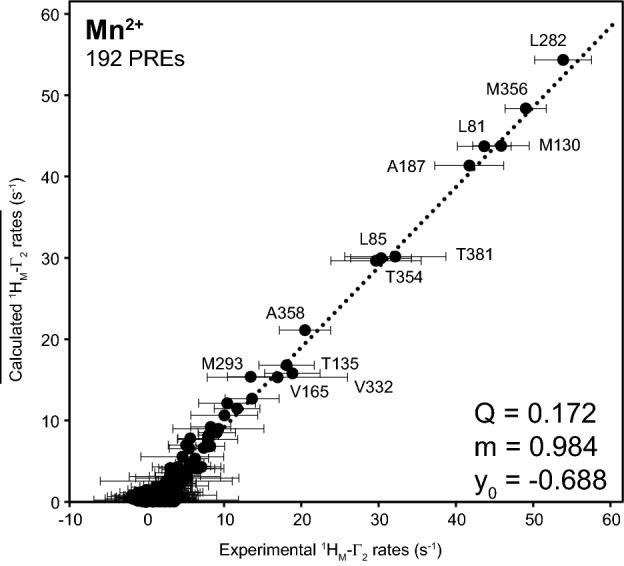
PREs confirm the location of the metal ion calculated from PCS. Correlation between observed and calculated values of side chain ^1^H_M_-Γ_2_ arising from UTP:Mn^2+^ complex bound to LmUGP. Calculated values represent the average for the “ensemble structure”. The experimental errors in the observed values are shown as horizontal bars. Residue numbers are indicated only for observable peaks showing the largest PREs

Fitting of the isotropic *g*-tensor requires preliminary knowledge of the rotational correlation times $\tau_{r}$ of the protein in solution, which was estimated from TRACT experiments (Fig. S10) (Lee et al. 2006). Similar to Δχ tensor fittings, the starting metal centre was extracted from the crystal structure with pdb 4M2A, but allowed to variate during the calculations. The best Q-factor (0.172) was observed for the “ensemble structure”, in line with the results obtained from PCS (see Fig. 9 and Table 2). Metal coordinates deviated < 0.5 Å from those obtained for lanthanide ions, indicating very similar binding mode to UTP during the interaction with LmUGP (Fig. 6a and Table S5). A complete list of observed and expected PREs can be found in Table S4. In addition, ^1^H,^13^C-HMQC spectra of MIL^proS^V^proS^AT LmUGP in the presence of UTP:Mn^2+^ and UTP:Mg^2+^ complexes, together with ^1^H_M_-Γ_2_ PRE rates can be scrutinized in Fig S9. These results were considered as indicators of correctness of the assignment. Nevertheless, such criteria only apply to methyl groups located 10 to 30 Å away from the metal binding site. Since most of the unassigned methyl groups are located at the periphery of the protein, only two additional signals could be assigned, namely M293 and M495. At this point 187 out of 199 methyl signals were assigned (94%), leaving 8 alanine and 4 threonine isolated methyl groups which were assigned by directed mutagenesis following standard approaches. For more information see Materials and Methods, Table S3 and Fig. S11.

A list of chemical shifts for MIL^proS^V^proS^AT methyl-labeled LmUGP in the UDP-Glc bound state can be found in the supplementary material (Table S6). ^1^H,^13^C HMQC spectra showing the complete assignment can be found in Fig S12 and S13 in the supplementary material, respectively.

## Conclusions

We have achieved a complete assignment of MIL^proS^V^proS^AT methyl-labeled samples of LmUGP in the presence and absence of UDP-Glc using a synergic combination of methyl-methyl NOESY experiments, paramagnetic NMR and directed mutagenesis. Noteworthy, neither NOEs nor paramagnetic NMR alone delivered enough information for a complete methyl side chain assignment. The acquisition of 4D HMQC-NOESY-HMQC spectra from two structurally different protein states has been instrumental to obtain the largest portion of the assignment. Protein structural rearrangements induced by ligand binding delivered unique sets of NOEs signals in each protein state. Signal overlapping was resolved owing to the different chemical shifts observed along the different enzyme states. Assignments could be transferred among protein states with simple ligand titrations. PCS were fundamental to expand the assignment beyond the limits imposed by 4D HMQC-NOESY-HMQC data alone, PREs allowed for independent validation of the assignments and directed mutagenesis permitted the assignment of isolated methyl groups. In short, this work provides new avenues for the assignment of large, methyl-labeled proteins. The complete assignment of LmUGP will also serve as basis for novel studies into the biological function of this important class of enzymes.

## Supplementary Information

Below is the link to the electronic supplementary material.

Supplementary file1 (PDF 3873 kb)

## Data Availability

Chemical shift assignments for MIL^proS^V^proS^AT methyl-labeled UDP-glucose pyrophosphorylase from *Leishmania major* have been deposited in the BioMagResBank (http://www.bmrb.wisc.edu) under the Accession Number 50749.

## Code availability

*Matlab* scripts used for data analysis are available from the corresponding author upon request.
