# Supplementary material for: Ligand-induced structural transitions combined with paramagnetic ions facilitate unambiguous NMR assignments of methyl groups in large proteins
Source: J Biomol NMR. 2022 Apr 10;76(3):59–74. doi: 10.1007/s10858-022-00394-0 (PMC9247001; doi:10.1007/s10858-022-00394-0)
Supplement: Supplementary file 1 — Supplementary file1 (PDF 3873 kb) [file 10858_2022_394_MOESM1_ESM.pdf]

Supplementary Information

Journal of Biomolecular NMR

Ligand-induced structural transitions combined with paramagnetic ions facilitate unambiguous NMR assignments of methyl groups in large proteins

Lars Mühlberg[1], Tuncay Alarcin[1], Thorben Maass[1], Robert Creutznacher,[1] Richard Küchler[1], and Alvaro Mallagaray[1*]

[1]Institute for Chemistry and Metabolomics, Center for Structural and Cell Biology in Medicine, University of Lübeck, Ratzeburger Allee 160, 23562 Lübeck, Germany

Corresponding author: Alvaro Mallagaray

Email: alvaro.mallagaraydebenito@uni-luebeck.de

Table of Contents:

Table S1: Sequence of primers used for the generation of single-point mutants.........................3

Preparation of culture media for the expression of [U-^{2}H], [^{13}C,^{1}H$_3$]-methyl labeled LmUGP......3

Table S2: Precursors and quantities used for the different [^{13}C,^{1}H$_3$]-methyl labeling schemes of LmUGP. 4

Table S3: List of samples and NMR experiments acquired in this study 5

Fig. S1: LmUGP binds metals in the presence of UTP 7

Fig. S2: Isotope labelling incorporation was uniform for all amino six amino acid types.............7

Fig. S3: Determination of optimal cut-off distance for *MAP-XSII* calculations when evaluating each LmUGP state independently 8

Fig. S4: Sequential titration of UTP and $MgCl_2$ into LmUGP. 8

Four-states binding model describing the interaction of LmUGP with UTP and a metal ion........9

Fig. S5: Measurement of K_Ds for the coordination of UTP with divalent and trivalent metals ...10

Fig. S6: Titration of MIL^{proS}V^{proS}AT methyl-labeled LmUGP with UTP 11

Fig. S7: The "ensemble structure" 11

Table S4: Experimental and calculated PCS and ^{1}H$_M$-Γ_2 values used in this study 12

Table S5: Final magnetic susceptibility tensors from proton PCS and metal coordinates obtained from PREs...16

Fig. S8: Determination of optimal distance for *MAP-XSII* calculations including PCS 17

Fig. S9: Analysis of PREs 18

Fig. S10: TRACT is used to estimate protein rotational correlation times τ_r 19

Fig. S11: Assignments obtained via mutagenesis 20

Table S6: ^{1}H and ^{13}C chemical shifts of the apo and UDP-Glc bound conformations of MIL^{proS}V^{proS}AT methyl-labeled LmUGP 23

Fig. S12: Complete assignment of the apo state of MIL^{proS}V^{proS}AT LmUGP 28

Fig. S13: Complete assignment of the UDP-Glc bound conformation of MIL^{proS}V^{proS}AT LmUGP.29

References 30

Table S1 Sequence of primers used for the generation of single-point mutants. Primers were purchased from Eurofins.

**Mutant**	**Primer sequence (5'→3')**
T96S	for: GCCGAGAACCCGG**GC**TACGAGTGGGCGC<br>rev: GCGCCCACTCGTA**GC**CCGGGTTCTCGGC
A145G	for: CTAGCACCAAGAGCTTTCTGAAGG**GC**CGCTACCCGTGGCTGTACCAGGTC<br>rev: GACCTGGTACAGCCACGGGTAGCG**GC**CCTTCAGAAAGCTCTTGGTGCTAG
T172S	for: CAAAATACTGCAGGACA**GC**CTCGAGCCGGCG<br>rev: CGCCGGCTCGAG**GC**TGTCCTGCAGTATTTTG
A183G	for: GCCGAGAACCCGG**GC**TACGAGTGGGCGC<br>rev: GCGCCCACTCGTA**GC**CCGGGTTCTCGGC
T226S	for: CCTCGGCGCCA**G**CATCGACAAGCGCGTG<br>rev: CACGCGCTTGTCGATG**C**TGGCGCCGAGG
A291G	for: GCCCAGTGCCCCAAGG**GC**GACATGGAAAGCTTCC<br>rev: GGAAGCTTTCCATGTC**GC**CCTTGGGGCACTGGGC
A345G	for: CGATTCATCTAATTCAGGCTCACCCAAGGTGTATC<br>rev: GATACACCTTGGGTGA**GC**CTGAATTAGATGAATCG
V413I	for: CGGCCACCCGCCT**A**T**T**GTTGACCTCGACAG<br>rev: CTGTCGAGGTCAAC**A**A**T**AGGCGGGTGGCCG
A419G	for: GTTGACCTCGACAGCG**GC**CACTACAAGATGATG<br>rev: CATCATCTTGTAGTG**GC**CGCTGTCGAGGTCAAC
A454G	for: CTGGTTCAGTTCGGTG**GC**GGCAACGTGCTC<br>rev: GAGCACGTTGCC**GC**CACCGAACTGAACCAG
A470G	for: GAGAACACGGACAGCG**G**CTCGGCGTTTGTG<br>rev: CACAAACGCCGAG**C**CGCTGTCCGTGTTCTC
T492S	for: CACCGCAGCAGTCGA**G**CAACAAGATGCGGC<br>rev: GCCGCATCTTGTTG**C**TCGACTGCTGCGGTG
M495I	for: CAGTCGACCAACAAGAT**T**CGGCCGCTCGAGC<br>rev: GCTCGAGCGGCCG**A**ATCTTGTTGGTCGACTG

Preparation of culture media for the expression of [*U*-^{2}H], [^{13}C,^{1}H$_3$]-methyl labeled LmUGP

The composition of these media have been described elsewhere.[1] An adapted version for the expression of LmUGP is reproduced here for clarity:

M9$^+$/D$_2$O minimal medium (for 100 mL): Mix 1.3 g Na_2HPO_4*2H_2O, 0.36 g anhydrous KH_2PO_4, 0.1 g NaCl, 0.3 g deuterated D-glucose (1,2,3,4,5,6,6-d$_7$) and 0.3 g NH_4Cl in 10 ml D_2O and lyophilize. Prepare another solution containing 46.5 mg of $MgSO_4$, 20 mg of $MgCl_2$, 1.42 mg of $CaCl_2$ and 2 mg of vitamin B1 in 5 ml D_2O and lyophilize. Prepare the 100x vitamins solution in 10 ml D_2O, which contains 0.1 mg of riboflavin and 1 mg of each of the following compounds: D-biotin, choline chloride, folic acid, nicotinamide, D-pantothenic acid, pyridoxal hydrochloride and cobalamine, all obtained from Aldrich. Dissolve the lyophilized powders and add 1 ml of the 100x vitamins solution in 99 ml D_2O. Adjust pH* to 7.50, add 100 µg/ml ampicillin and filter under sterile conditions. The medium can be stored at -20 °C for a week.

M9$^+$/D$_2$O minimal medium containing the isotopically labeled precursors (for 10 mL): Mix 130 mg Na_2HPO_4*2H_2O, 36 mg anhydrous KH_2PO_4, 10 mg NaCl, 30 mg deuterated D-glucose (1,2,3,4,5,6,6-d$_7$), 30 mg NH_4Cl, 4.65 mg of $MgSO_4$, 2 mg of $MgCl_2$, 0.14 mg of $CaCl_2$ and 0.2 mg of vitamin B1 in 5 ml D_2O and lyophilize. Mix the lyophilized powders, the desired labeled amino acids or precursors (Table S2) and 10 µl of the 100x vitamins solution and add D_2O up to 10 ml. Adjust pH* to 7.50, add 100 µg/ml ampicillin and filter under sterile conditions. The solution can be preserved at -20 °C for a week.

Table S2 Precursors and quantities used for the different [^{13}C,$^{1}H_3$]-methyl labeling schemes of LmUGP. A systematic review on precursors required for optimal methyl group isotope labelling in *E. coli* can be found somewhere else.[2]

Labeled amino acid	Precursor	Quantity (mg/L)	Remarks
Met-ε	L-Methionine (6-^{13}C)[a]	250	Non-deuterated Met.[3]
Ile-δ1	2-ketobutyric acid (4-^{13}C;3,3-D_2)	60	First described in 1997.[4] Prepared from 2-keto-4-^{13}C-butyric acid (CortecNet).[1]
Leu-δ2, Val-γ2 pro-(*S*)	2-hydroxy-2-(^{13}C)methyl-3-oxobutanoate (4-D_3)[b]	120	No scrambling observed.[5]
Val-γ2 pro-(*S*)	2-hydroxy-2-(^{13}C)methyl-3-oxobutanoate (4-D_3)[b]<br>L-Leucine (D_{10})[c]	120<br>80	Addition of Leucine-d_{10} was required to inhibit the use of 2-acetolactate in leucine biogenesis.[6]
Ala-β	L-Alanine (3-^{13}C,2-D)[d]<br>Succinate-d_4[a]	400<br>2,540	2.5 – 5% scrambling into Leu and Val[7]
Thr-γ	L-Threonine (4-^{13}C;2,3-D_2)[e]<br>L-Glycine (D_5)[e]	50<br>100	Ile-δ1 needs to be labeled simultaneously to Thr-γ to prevent scrambling.[8]

Precursors and amino acids were obtained from [a]Aldrich, [b]NMR-Bio, [c]CortecNet, [d]Eurisotop and [e]Cambridge Isotope Laboratories (CIL).

Table S3 List of samples and NMR experiments acquired in this study.

N°	Labeling scheme: [*U*-^{15}N,^{2}H] +	LmUGP conc. (µM)	Construct	Buffer[a]	Metals and ligands	NMR experiment	Data points in direct and indirect dimensions	SW in direct and indirect dimensions (ppm) or NUS	Number of scans, recovery delay (s)	Magnetic field (MHz)
1	MIL^{proS}V^{proS}AT	450	Wild type	A	5 mM $MgCl_2$ (apo state)	4D HMQC-NOESY-HMQC	512(^{1}H)x70(^{13}C) x84(^{1}H)x52(^{13}C)	30% NUS (11,466 complex points), 180 ms mixing time	4, 1	900
2	MIL^{proS}V^{proS}AT	450	Wild type	A	5 mM $MgCl_2$ + 12 mM UDP-glucose (UDP-Glc bound state)	4D HMQC-NOESY-HMQC	512(^{1}H)x66(^{13}C) x80(^{1}H)x52(^{13}C)	31.36% NUS (10,764 complex points), 180 ms mixing time	4, 1	950
3	MIL^{proS}V^{proS}AT	110	Wild type	A	10 mM $MgCl_2$, titration of UTP	^{1}H,^{13}C HMQC	1024x512	7.5x19	4, 1.5	500
4	MIL^{proS}V^{proS}AT	154	Wild type	A	10 mM $MgCl_2$, titration of UDP-glucose	^{1}H,^{13}C HMQC	1024x512	7.5x19	4, 1.5	500
5	MIL^{proS}V^{proS}AT	100	Wild type	B	3.5 mM UTP + 2.1 mM $LuCl_3$/$LaCl_3$/$CeCl_3$/$EuCl_3$/ $TbCl_3$/$TmCl_3$	^{1}H,^{13}C HMQC	512x256	3.7x19	4, 1.5	500
6	MIL^{proS}V^{proS}AT	199	Wild type	B	1.5 mM UTP + 55 µM $MgCl_2$/$MnCl_2$	^1H_M-Γ_2 PRE relaxation rates	512x512	4.2x19	4, 1.5	600
7	M	267	Wild type	A	5 mM $MgCl_2$	^{1}H,^{13}C HMQC	1024x1024	7.5x19	4, 1.5	500
8	I	200	Wild type	A	5 mM $MgCl_2$	^{1}H,^{13}C HMQC	1024x1024	7.5x19	4, 1.5	500
9	L^{proS}V^{proS}	312	Wild type	A	5 mM $MgCl_2$	^{1}H,^{13}C HMQC	1024x1024	7.5x19	4, 1.5	500
10	V^{proS}	250	Wild type	A	5 mM $MgCl_2$	^{1}H,^{13}C HMQC	1024x1024	7.5x19	4, 1.5	500
11	A	250	Wild type	A	5 mM $MgCl_2$	^{1}H,^{13}C HMQC	1024x1024	7.5x19	4, 1.5	500
12	MIL^{proS}V^{proS}AT	450	Wild type	A	5 mM $MgCl_2$	^{1}H,^{13}C HMQC	1024x1024	7.5x19	4, 1.5	500

[a]For details see “NMR sample preparation” in main manuscript.

Table S3 (continuation).

N°	**Labeling scheme: [*U*-^{15}N,^{2}H] +**	**LmUGP conc. (µM)**	**Construct**	**Buffer[a]**	**Metals and ligands**	**NMR experiment**	**Data points in direct and indirect dimensions**	**SW in direct and indirect dimensions (ppm)**	**Number of scans, recovery delay (s)**	**Magnetic field (MHz)**
13	A<br>$L^{proS}V^{proS}$<br>M	40 (3 species)	A419G<br>V413I<br>M495I	A	10 mM $MgCl_2$ (apo), then + 4 mM UDP-glucose (UDP-Glc bound)	^{1}H,^{13}C HMQC	512x512	3.7x18 or 3.7x10 ppm for Ala region	32, 1.5	600
14	A<br>T	110 (2 species)	A291G<br>T172S	A	10 mM $MgCl_2$ (apo), then + 4 mM UDP-glucose (UDP-Glc bound)	^{1}H,^{13}C HMQC	512x512/1024	3.7x18	16, 1.5	600
15	A<br>T	67 (2 species)	A454G<br>T96S	A	10 mM $MgCl_2$ (apo), then + 4 mM UDP-glucose (UDP-Glc bound)	^{1}H,^{13}C HMQC	512x512	3.7x18	4, 1.5	600
16	A<br>T	100 (2 species)	A470G<br>T226S	A	10 mM $MgCl_2$ (apo), then + 4 mM UDP-glucose (UDP-Glc bound)	^{1}H,^{13}C HMQC	512x512	3.7x18	8, 1.5	600
17	A<br>T	177 (2 species)	A345G<br>T492S	A	10 mM $MgCl_2$ (apo), then + 4 mM UDP-glucose (UDP-Glc bound)	^{1}H,^{13}C HMQC	512x512	3.7x18	8, 1.5	600
18	A	105	A145G	A	10 mM $MgCl_2$ (apo), then + 4 mM UDP-glucose (UDP-Glc bound)	^{1}H,^{13}C HMQC	512x512	3.7x18	4, 1.5	600
19	A	26	A183G	A	10 mM $MgCl_2$ (apo), then + 4 mM UDP-glucose (UDP-Glc bound)	^{1}H,^{13}C HMQC	512x512	3.7x18	16, 1.5	600
20	V^{proS}	50 - 335	Wild type	A	5 mM $MgCl_2$	^{1}H,^{15}N TRACT	2048	16	16-128, 2	500, 600
21	$MIL^{proS}V^{proS}AT$	154	Wild type	A	10 mM $MgCl_2$, titration of UDP-glucose	^{1}H,^{13}C HMQC	1024x512	7.5x19	4, 1.5	500
22	$MIL^{proS}V^{proS}AT$	80 - 110	Wild type	A	No metal or 10 mM $MgCl_2$ or 5 mM $LnCl_3$, titration of UTP	^{1}H,^{13}C HMQC	1024x512	7.5x19	4, 1.5	500
23	-	-	-	A/B	UTP at 239.2 or 250 µM, titration of $MgCl_2$, $LaCl_3$, $LuCl_3$, $EuCl_3$ and $CeCl_3$	^{1}H NMR	32768	11.6	16, 3	600

[a]For details see “NMR sample preparation” in main manuscript

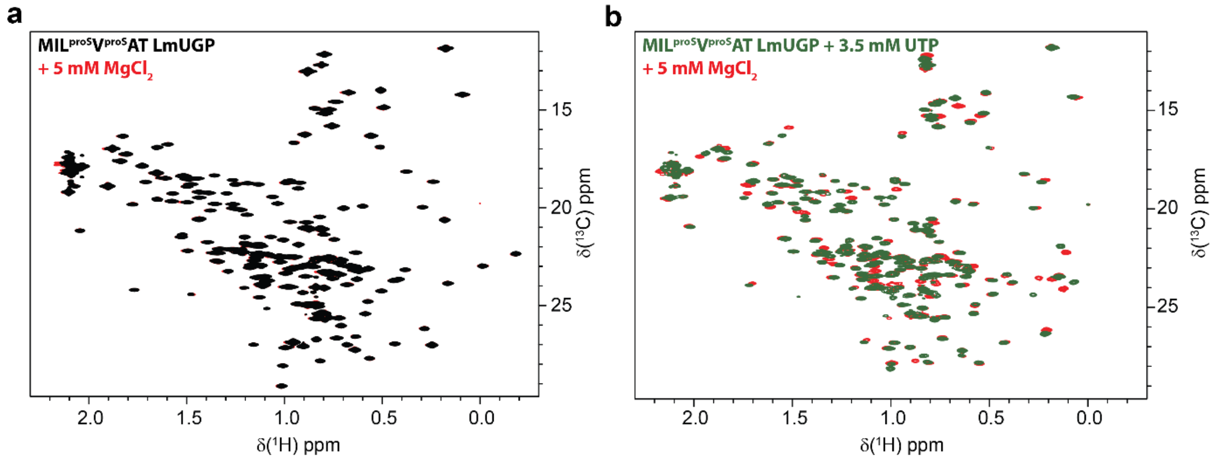

Fig. S1 LmUGP binds metals in the presence of UTP. Superimposition of ^{1}H,^{13}C HMQC spectra of a MIL^{proS}V^{proS}AT LmUGP sample acquired **a** in the absence (black) and presence (red) of 5 mM $MgCl_2$, and **b** in the presence of 3.5 mM UTP (green) and after the addition of 5 mM $MgCl_2$. (red). Samples from (a) and (b) contained 140 µM and 80 µM protein concentration, respectively. All samples were prepared in buffer B, and experiments were acquired at 293 K and 500 MHz.

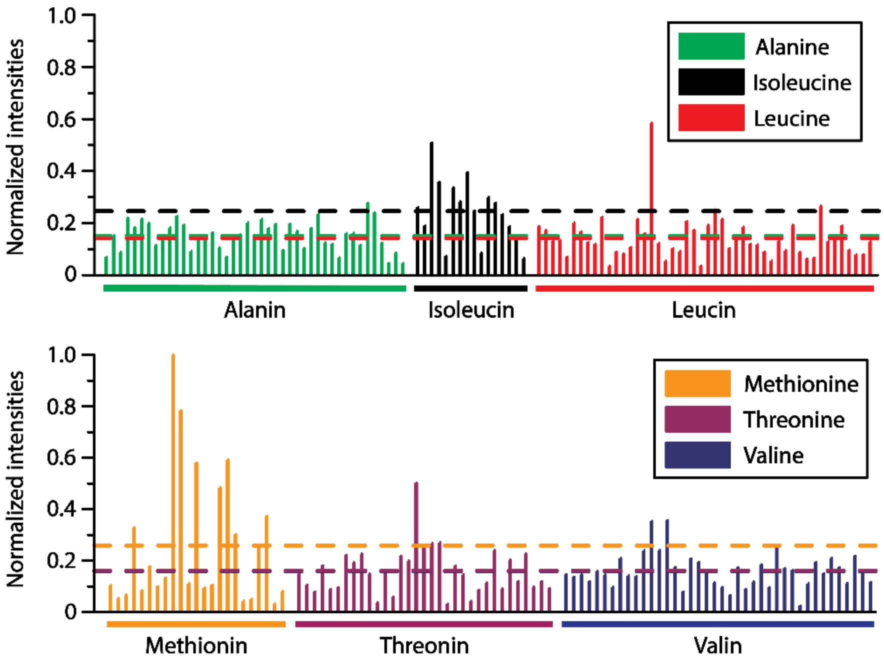

Fig. S2 Isotope labelling incorporation was uniform for all amino six amino acid types. Relative peak intensities extracted from a ^{1}H,^{13}C HMQC spectrum of MIL^{proS}V^{proS}AT LmUGP in the apo state. Intensities were normalized according to the signal showing the largest absolute intensity (Met 5). Shaded lines indicate the average normalized intensity of the corresponding amino acid type (valine and threonine show identical average intensity). Data extracted using a sample containing 450 µM protein concentration. NMR experiment was acquired at 293 K and 900 MHz.

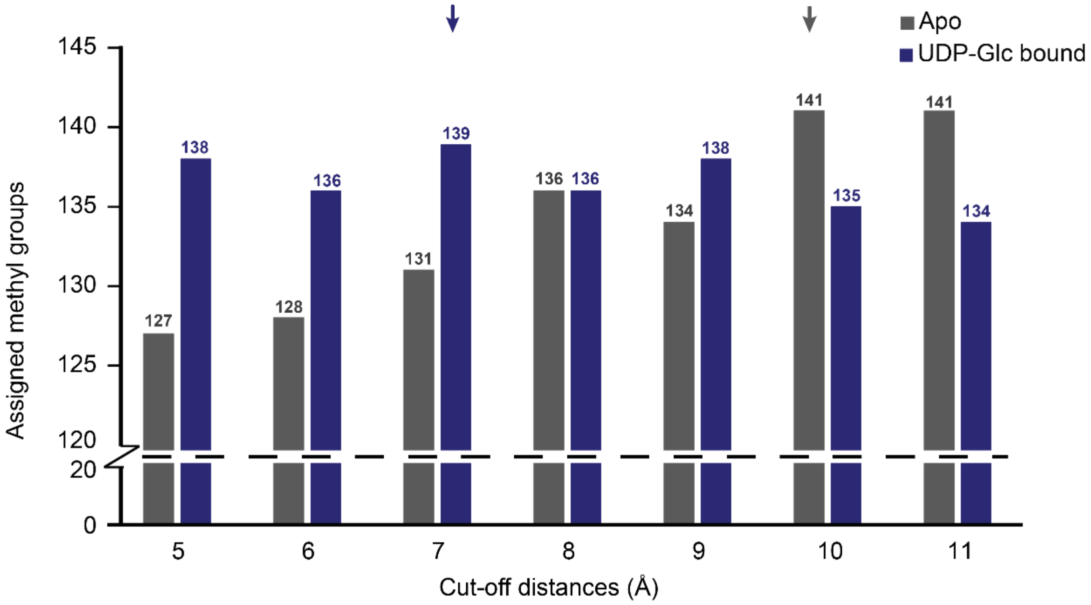

Fig. S3 Determination of optimal cut-off distance for *MAP-XSII* calculations when evaluating each LmUGP state independently. 20 MMC trials with *MAP-XSII* were performed for independently each state with cut-off distances varying from 5 to 11 Å. Gray corresponds to apo state and blue indicates UDP-Glc bound state. Signals consistently assigned to the same residue were considered as "assigned", and correspond to the number indicated over each bar. Selected cut-off distances for each protein conformation are indicated with an arrow.

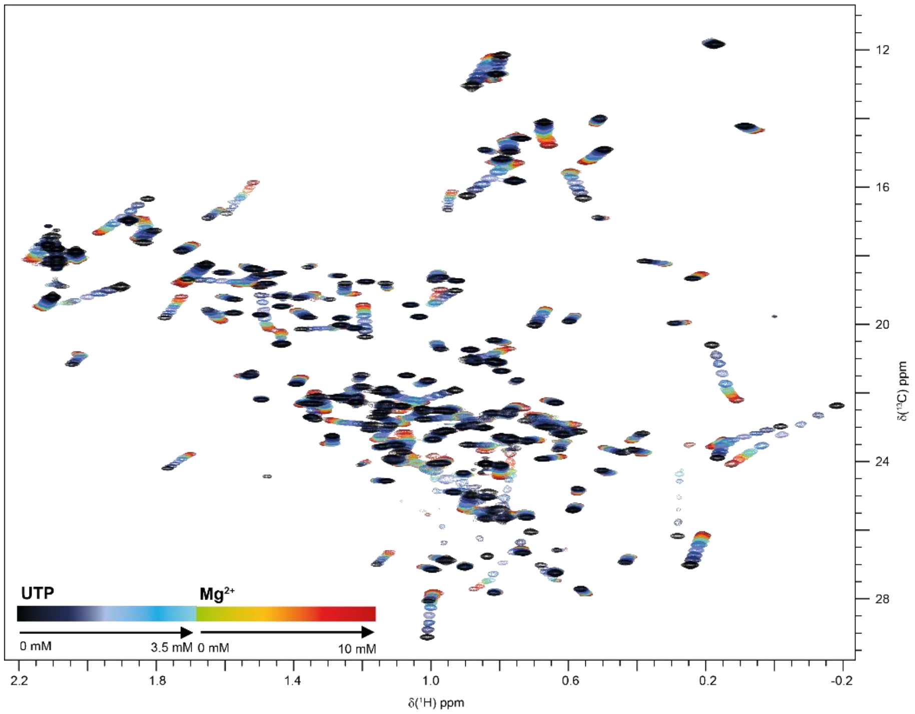

Fig. S4 Sequential titration of UTP and $MgCl_2$ into LmUGP. Superimposition of $^1H,^{13}C$ HMQC spectra of a $MIL^{proS}V^{proS}AT$ LmUGP sample acquired for the titration of UTP up to 3.5 mM followed by the titration of $MgCl_2$ up to 10 mM. Spectra were acquired at 293 K and 500 MHz.

Four-states binding model describing the interaction of LmUGP with UTP and a metal ion:

In the four-states binding model depicted in Fig. 7, dissociation constants K_{D1-4} are described as (Eq. 1):

$$K_{D1} = \frac{[U][M]}{[UM]};\ K_{D2} = \frac{[U][P]}{[UP]};\ K_{D3} = \frac{[UM][P]}{[UMP]};\ K_{D4} = \frac{[UP][M]}{[UMP]} \quad (1)$$

where K_{D1} corresponds to the association of UTP and the metal, K_{D2} to the binding of UTP to the protein, K_{D3} to the binding of the complex UTP:metal to the protein, and K_{D4} to the binding of the metal to UTP in complex with the protein. [U], [P], [M] are the concentrations of the free UTP, protein and metal; [UM], [UP] and [UMP] correspond to the concentrations of the complexes UTP:metal, UTP:Protein and UTP:Metal:Protein, respectively. They are related to the total UTP, protein and metal concentration ($[U]_t$, $[P]_t$ and $[M]_t$) as described in Eq. 2:

$$[M]_t = [M] + [UM] + [UMP]$$
$$[P]_t = [P] + [UP] + [UMP]$$
$$[U]_t = [U] + [UM] + [UP] + [UMP] \quad (2)$$

Substitution of Eq. 2 in Eq. 1 renders the system of nonlinear equations Eq. 3. K_{D1-3} can be obtained from titrations under the adequate experimental conditions using a simple two states model (Eq. 6 and 7 from Materials and Methods). This yields a system of four nonlinear equations with four variables ([UM], [UP], [UMP] and K_{D4}), which can be approximated by nonlinear least-squares minimization. We selected the Levenberg-Marquart algorithm with 10,000 maximum number of iterations and a termination tolerance on the function value of $1e^{-11}$, as implemented in the *fsolve* function in Matlab R2019b package.

$$\frac{([U]_t-[UM]-[UP]-[UMP])([M]_t-[UM]-[UMP])}{[UM]} - K_{D1} = 0$$
$$\frac{([U]_t-[UM]-[UP]-[UMP])([P]_t-[UP]-[UMP])}{[UP]} - K_{D2} = 0$$
$$\frac{[UM]([P]_t-[UP]-[UMP])}{[UMP]} - K_{D3} = 0$$
$$\frac{[UP]([M]_t-[UM]-[UMP])}{[UMP]} - K_{D4} = 0 \quad (3)$$

Errors in the fittings were determined from a Monte Carlo approach with 1,000 iterations as previously described.[11] Errors are given as one standard deviation.

Under the experimental conditions used in this study, the concentration of free paramagnetic and diamagnetic trivalent metals remained always below 60 µM (max: 53.0 µM for La^{3+} – min: 2.9 µM for Mn^{2+}). No effects due to unspecific metal binding to the protein could be observed at such low concentrations. Thus, any paramagnetic effect observed in the [^{13}C,^{1}H]-HMQC spectra can be attributed to the fraction of the protein bound to UTP and metal f_{UMP}, which is described by Eq. 4. Therefore, f_{UMP} was used to weight the calculated Γ_2 as described in Material and Methods.

$$f_{UMP} = \frac{[UMP]}{[P]_t} \quad (4)$$

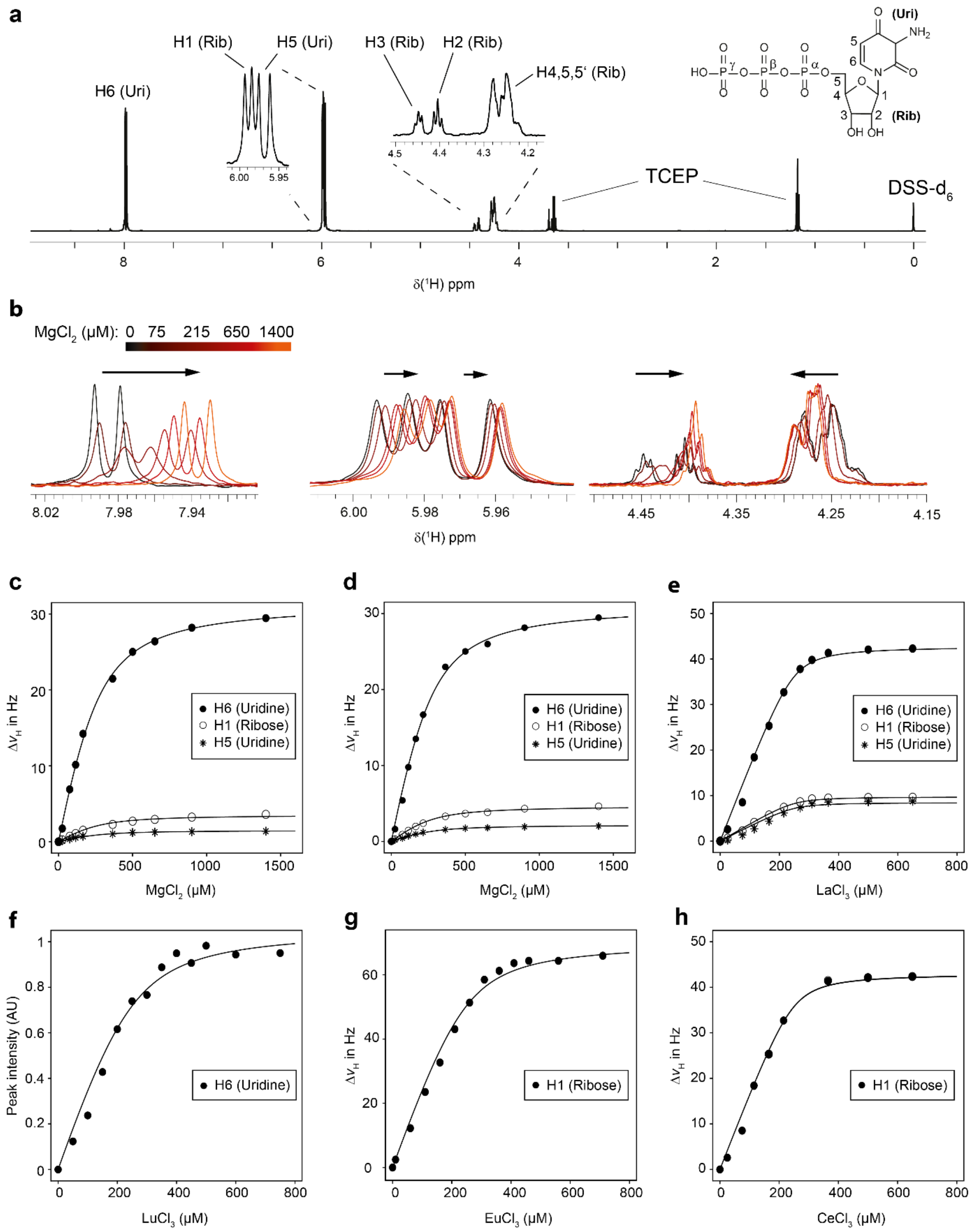

Fig. S5 Measurement of K_Ds for the coordination of UTP with divalent and trivalent metals. **a** ^{1}H NMR spectrum of UTP in at pH* 7.06 showing the assignment. **b** Titration of $MgCl_2$ produces clear CSP in the UTP signals H_5/H_6 and H_1 from the uridine and ribose moiety, respectively. CPS and change in signal intensity indicate binding, which can be utilized to extract dissociation constants K_D. Only isolated signals were selected and fitted to Eq. 9. Binding isotherms were obtained for the following di- and trivalent metals: **c** and **d** correspond to $MgCl_2$ at pH* 7.20 and 7.06, respectively. **e, f, g** and **h** were titrated at pH* 7.06 with $LaCl_3$, $LuCl_3$, $EuCl_3$ and $CeCl_3$, respectively. The sample corresponding to (a) was prepared in buffer A, and all other titrations were performed in buffer B, as explained Materials and Methods. NMR experiments acquired at 600 MHz and 293 K.

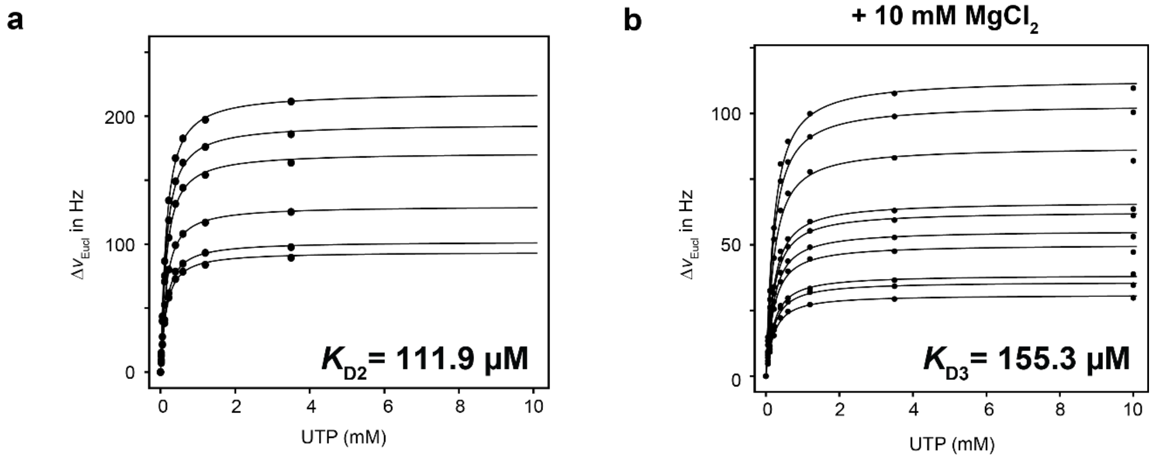

Fig. S6 Titration of MIL^{proS}V^{proS}AT methyl-labeled LmUGP with UTP **a** in the absence and **b** in the presence of an excess of $MgCl_2$. CSP were calculated as Euclidean distances according to Eq. 7. Only signals showing CSP larger than *mean + 2σ* in the ^1H,^{13}C HMQC spectra at the highest UTP concentration were subjected to global fitting to the law of mass action (Eq. 9). Experiments were acquired at 500 MHz and 293 K.

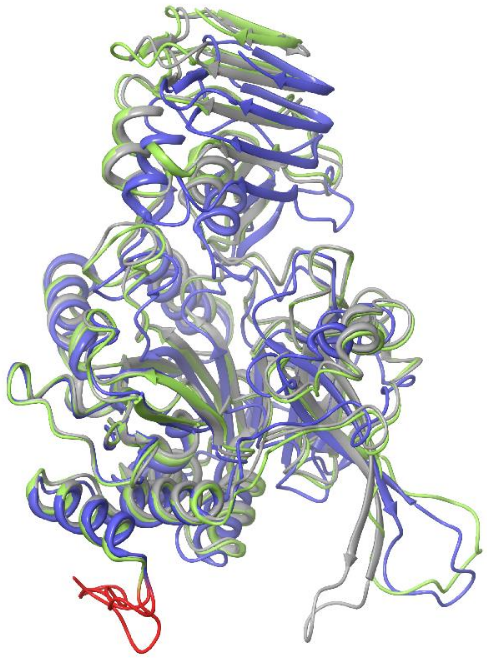

Fig. S7 The "ensemble structure". Superimposition of crystal structures with pdb 2OEF (apo, grey), 4M28 (dUpCpp bound, green) and 4M2A (UDP-Glc bound, blue) used for $\Delta\chi$ tensor and PREs fittings. Crystal structures were superimposed as described in Führing et al.[9] *N*-terminus was modelled for each conformer using MoodLop,[10] and is indicated as a red cartoon.

Table S4 Experimental and calculated PCS and ^1H_M-Γ_2 values used in this study. Calculated values correspond to the "ensemble structure" (pdb codes 2OEF, 4M2A and 4M28).

PCS												^1H_M-Γ_2			
Ce^{3+}			Eu^{3+}			Tm^{3+}			Tb^{3+}						
**Amino acid**	**Exp (Hz)**	**$Calc_{aver}$ (Hz)**	**Amino acid**	**Exp (Hz)**	**$Calc_{aver}$ (Hz)**	**Amino acid**	**Exp (Hz)**	**$Calc_{aver}$ (Hz)**	**Amino acid**	**Exp (Hz)**	**$Calc_{aver}$ (Hz)**	**Amino acid**	**Exp (s^{-1})**	**Exp error (s^{-1})**	**$Calc_{aver}$ (s^{-1})**
**1**	-4.5	-3	**1**	2	0	**8**	3.5	2.5	**1**	-24.5	-9.5	**1**	0.9	1.0	0.1
**5**	-4	-3	**5**	1.5	1	**10**	-4	-3.5	**5**	-24	-11	**5**	1.0	0.6	0.1
**8**	-7	-7	**10**	2	1.5	**11**	-4.5	-2.5	**8**	-41	-41.5	**8**	-	-	0.3
**10**	-2	-3	**11**	3.5	3.5	**12**	1.5	1.5	**10**	-4	-8.5	**10**	2.0	4.0	0.1
**11**	-4.5	-5.5	**12**	6	3.5	**14**	-9.5	-5.5	**11**	-19.5	-22	**11**	1.2	1.9	0.4
**12**	-5.5	-7	**14**	0.5	0.5	**16**	-1	2	**12**	-37	-40	**12**	-1.9	1.7	0.4
**14**	-0.5	-2.5	**16**	-2	2	**22**	-7	-8	**14**	13	2	**14**	3.5	1.4	0.3
**16**	-9.5	-6.5	**19**	0.5	1.5	**27**	14.5	16	**16**	-34.5	-34.5	**16**	-1.7	3.7	0.4
**19**	-12.5	-11.5	**24**	1	-3	**29**	5	4	**22**	45	33.5	**19**	1.2	3.2	1.8
**22**	3.5	1	**29**	2	1	**33**	5	4.5	**29**	-31.5	-36	**22**	3.6	2.5	0.6
**24**	-4.5	-4	**31**	9.5	10	**34**	9	13	**33**	-49	-51.5	**24**	3.8	3.4	1.2
**27**	-8.5	-13	**33**	1.5	3.5	**37**	4.5	5	**37**	-60	-60.5	**27**	2.5	4.2	0.9
**29**	-7	-6.5	**34**	5.5	6.5	**38**	10	7.5	**38**	-89	-82	**29**	0.3	1.5	0.5
**31**	-27	-26	**37**	5.5	5	**44**	9.5	10.5	**51**	38	38.5	**31**	4.4	2.5	3.9
**34**	-14	-13.5	**38**	9.5	7.5	**51**	-14	-12.5	**53**	66.5	66	**33**	-1.3	3.3	0.8
**37**	-9.5	-9	**39**	20	19.5	**53**	-16	-15.5	**64**	59.5	63	**34**	-1.2	2.3	1.1
**38**	-12	-10.5	**44**	22.5	17	**55**	-35.5	-35	**65**	38	38	**37**	2.9	1.0	0.5
**39**	-23.5	-25.5	**47**	-6	-4	**58**	-32	-31	**66**	28	32	**38**	3.9	2.7	1.2
**44**	-19	-17	**51**	0	-1	**60**	-21.5	-23	**71**	0	0	**39**	7.1	2.5	4.3
**47**	16	16.5	**52**	-10	-11.5	**61**	-23	-21	**72**	6	8	**44**	5.1	0.7	3.2
**52**	17	20.5	**53**	-4.5	-4.5	**64**	-15.5	-15.5	**73**	-7	-4	**47**	5.6	1.6	7.8
**53**	7.5	7	**55**	-14	-14	**65**	-10	-11.5	**76**	-23	-20	**51**	-1.9	0.9	0.8
**55**	20	22	**58**	-10.5	-12	**66**	-9.5	-10	**78**	-50.5	-50	**52**	3.1	1.0	2.1
**58**	17.5	18.5	**60**	-6	-7.5	**71**	-3	-4.5	**112**	-38.5	-41	**53**	-0.8	1.3	0.2
**60**	10.5	11	**61**	-6	-5	**72**	-6.5	-6	**117**	-16.5	-12.5	**55**	2.7	1.0	0.9
**61**	9	9	**64**	-4	-4	**73**	-4.5	-4.5	**125**	-41	-36	**58**	-1.7	2.2	0.6
**64**	6.5	6.5	**65**	-2	-2	**76**	-1	-1	**137**	54.5	51	**60**	0.2	1.8	0.3
**65**	4	3.5	**66**	-1.5	-2	**77**	6	6.5	**145**	-18.5	-18.5	**61**	3.9	1.6	0.4
**66**	1	3	**71**	0.5	0.5	**78**	5	3	**153**	-52	-43	**64**	2.4	2.3	0.1
**71**	-1	-1	**72**	-1.5	-0.5	**97**	2	7	**172**	6.5	-3	**65**	1.7	2.9	0.1
**72**	0	0	**73**	0.5	1.5	**100**	13	16.5	**208**	-35	-40	**66**	0.7	3.1	0.0
**73**	-1.5	-1.5	**76**	1.5	2	**105**	3.5	6	**227**	-9.5	-19.5	**71**	2.3	3.3	0.0
**76**	-4.5	-2.5	**77**	5	3.5	**107**	18	16.5	**232**	17	14.5	**72**	2.9	2.6	0.1
**77**	-6	-7	**78**	4	5.5	**111**	9	6	**233**	30.5	30	**73**	0.5	1.6	0.2
**78**	-7.5	-7	**79**	20.5	19.5	**112**	0	1	**235**	36	43.5	**76**	-1.5	1.5	0.4
**79**	-37.5	-34.5	**89**	31	35	**117**	-7	-3.5	**240**	71	70	**77**	2.0	0.8	0.5
**89**	-44	-42.5	**94**	19	15	**125**	0	1	**263**	44.5	41.5	**78**	-1.1	1.9	0.8
**91**	-1	-1.5	**100**	23.5	23.5	**137**	-13.5	-11.5	**264**	35	32	**79**	8.2	1.9	6.8
**94**	-11	-11	**105**	9	12.5	**139**	19	14.5	**266**	21	21.5	**81**	43.7	3.5	43.7
**96**	-55.5	-55	**107**	18	16.5	**143**	8.5	7	**275**	24	25	**85**	30.3	3.9	30.0
**98**	-26.5	-18	**111**	12.5	9.5	**145**	-2	-2	**279**	62	65	**87**	-	-	128.4
**100**	-17.5	-24.5	**112**	5.5	6	**153**	3.5	3.5	**316**	3.5	-0.5	**89**	13.6	3.5	12.7
**105**	-10	-12	**117**	1.5	3	**158**	11	8.5	**318**	-33.5	-42.5	**91**	5.5	2.5	6.6
**107**	-19.5	-18.5	**125**	4.5	4.5	**161**	7.5	9	**320**	-17	-24	**94**	7.9	3.9	7.7
**110**	-22	-17.5	**128**	10	7.5	**168**	3.5	4	**369**	103.5	101	**96**	11.7	2.9	11.5
**111**	-8	-10.5	**135**	-5	0	**172**	-10.5	-6	**392**	-30.5	-29	**97**	2.1	1.2	1.8
**114**	-9	-10	**137**	-5.5	-3	**173**	1	1.5	**394**	-10.5	-11.5	**98**	6.6	2.2	4.1
**117**	-1.5	-3	**139**	10.5	12	**179**	-24	-19	**395**	-37.5	-41	**100**	4.0	2.3	2.2
**125**	-5.5	-5.5	**143**	8.5	9	**196**	24	33	**396**	-10.5	-6	**105**	2.8	1.5	1.0
**128**	-16	-15.5	**145**	3	3.5	**204**	10.5	11	**401**	-63.5	-71.5	**107**	2.2	1.2	1.8
**129**	-34.5	-30	**153**	3.5	4	**207**	7.5	7	**402**	-2	0	**110**	2.8	2.9	1.6
**135**	8	6	**158**	3.5	6	**208**	2	4	**403**	-2.5	3.5	**111**	-1.3	2.8	0.5
**137**	5.5	4.5	**160**	15.5	11.5	**215**	16	17	**431**	-54.5	-64.5	**112**	1.8	1.0	0.2

**139**	-17	-15
**143**	-11	-10.5
**145**	-2.5	-3.5
**153**	-7	-6.5
**158**	-11	-9.5
**160**	-31	-29.5
**161**	-10	-8
**165**	-25.5	-23
**168**	-16.5	-14
**169**	6.5	3
**172**	-1	-3
**173**	-13.5	-11
**179**	12	8
**183**	35.5	27
**194**	-36	-33
**196**	-24.5	-22.5
**197**	-35	-30
**198**	-17.5	-17
**204**	-8	-8.5
**207**	-9.5	-7.5
**208**	-5	-5
**215**	-13	-12.5
**217**	-6	-11.5
**225**	4	0.5
**226**	-3	-5.5
**227**	-1.5	-3
**231**	7.5	9
**232**	1.5	1.5
**233**	2.5	3
**235**	4	5.5
**240**	8.5	8.5
**243**	11.5	12
**244**	54.5	63
**246**	34.5	34.5
**250**	20.5	18
**259**	31.5	33.5
**260**	24	27.5
**263**	5.5	4.5
**264**	7	4
**266**	3	2.5
**275**	2.5	2.5
**280**	18.5	20.5
**281**	17	20.5
**291**	0	-5.5
**299**	14.5	14.5
**307**	55.5	57
**310**	42	45
**312**	-0.5	-2
**314**	-6.5	-7
**316**	0.5	0
**317**	-7.5	-8.5
**318**	-5	-5.5
**320**	1	-2
**321**	-13	-14
**327**	-10	-11.5
**328**	-4	-9
**332**	-39.5	-46
**345**	10.5	7.5
**352**	-46	-36
**356**	10.5	9
**358**	22.5	16

**165**	3	2.5
**168**	12.5	10
**169**	-2.5	-1
**172**	2	2.5
**173**	10	9.5
**176**	-9	-5
**177**	-4	-4.5
**179**	-8.5	-5.5
**183**	-25	-20.5
**194**	11.5	14.5
**196**	1.5	6.5
**197**	9.5	8.5
**198**	6	6.5
**204**	4	2.5
**207**	1	3.5
**208**	0	1.5
**215**	6.5	6
**217**	5	6
**225**	5.5	7
**226**	9	7
**227**	5.5	3
**231**	-7	-5
**232**	2	-1
**233**	-3.5	-1.5
**235**	-6	-4
**240**	-8	-5.5
**243**	-6.5	-7.5
**259**	-20.5	-21.5
**260**	-12	-16.5
**263**	-3	-3
**264**	-1	-2.5
**266**	1.5	-1.5
**275**	-1.5	-2
**279**	-4.5	-5.5
**280**	-13.5	-13
**281**	-8.5	-11.5
**291**	1	5
**299**	-11	-13
**314**	4	2.5
**316**	-2	-1
**317**	5	2
**318**	0	1.5
**320**	5.5	-0.5
**321**	8.5	4
**328**	3.5	1.5
**330**	-2.5	0
**332**	31.5	32
**338**	-7	-3.5
**345**	-6.5	-4
**349**	-6	-4.5
**359**	-9.5	-6
**360**	-18	-19.5
**361**	-9	-7.5
**362**	-5.5	-0.5
**368**	-16.5	-18.5
**369**	-6.5	-7
**370**	-9.5	-14
**371**	-13.5	-16
**377**	7	9
**379**	37.5	35.5
**383**	14	12.5

**223**	10.5	10
**225**	-13.5	-18
**226**	-6	-4
**227**	-2	-2
**231**	-22.5	-19.5
**232**	-7	-7.5
**233**	-11	-10
**235**	-11.5	-12
**240**	-18	-16.5
**243**	-23	-22
**263**	-12.5	-12
**266**	-9.5	-8.5
**275**	-8.5	-9
**279**	-16	-16
**280**	-32.5	-32.5
**281**	-27.5	-32
**299**	-28.5	-27
**312**	4.5	7
**314**	3	9
**316**	-4	-3.5
**317**	15.5	14
**318**	1	4.5
**320**	-3.5	1
**321**	19.5	20.5
**327**	7.5	13
**338**	-31.5	-30
**345**	-18	-18
**358**	-10.5	-11.5
**361**	-17	-17.5
**362**	2	0.5
**366**	-21	-19.5
**369**	-25	-22.5
**370**	-38	-36.5
**377**	-14	-13.5
**386**	14	9
**387**	8.5	6
**388**	8	7.5
**392**	-0.5	-1.5
**394**	-6	-4.5
**395**	-0.5	-0.5
**396**	-9.5	-6
**401**	2.5	2.5
**402**	-6	-7
**403**	-13	-7.5
**413**	-5.5	-3
**414**	11.5	7.5
**416**	17	12.5
**419**	16.5	10
**424**	1.5	11
**430**	11.5	9
**431**	6	4.5
**435**	4	2.5
**438**	2.5	2
**439**	0	-4
**444**	7	4
**445**	11	8
**446**	12.5	7
**449**	1	-0.5
**450**	3.5	3
**454**	-2	-2
**457**	-1.5	-2.5

**435**	-56	-51.5
**438**	-44	-41.5
**439**	-12	-8
**444**	-54.5	-49
**449**	-23.5	-22
**450**	-41	-40
**454**	-14	-18
**457**	-17.5	-14
**458**	-37	-35
**459**	-10.5	-7
**461**	-30.5	-18.5
**462**	-24	-20.5
**463**	-38	-37
**464**	-40	-37.5
**467**	-22	-23.5
**470**	0	-11.5
**472**	-18.5	-19
**474**	-16	-17
**475**	-29.5	-29
**479**	-17	-16.5
**481**	-20	-17
**484**	-17	-16
**485**	-24	-22.5
**486**	-22.5	-21
**492**	-8	-8
**495**	-10.5	-7
**498**	-10	-9.5

**114**	1.4	0.8	0.5
**117**	-0.7	1.4	0.2
**125**	0.0	1.4	0.3
**128**	3.2	3.7	1.1
**129**	3.0	2.0	3.6
**130**	45.8	3.7	43.8
**135**	18.1	3.6	16.8
**137**	2.4	2.5	0.9
**139**	2.8	2.0	1.9
**143**	2.1	1.8	0.6
**145**	2.6	2.7	0.2
**150**	2.4	4.0	0.3
**153**	1.3	2.6	0.2
**158**	1.1	1.5	0.5
**160**	6.2	3.8	4.1
**161**	2.1	2.5	2.0
**165**	16.9	9.1	15.3
**168**	3.1	2.0	3.1
**169**	3.8	1.4	1.1
**172**	1.1	2.6	0.3
**173**	1.6	1.9	1.5
**176**	2.9	1.3	1.7
**177**	3.6	1.9	0.5
**179**	2.4	4.2	0.2
**183**	2.9	2.2	2.0
**187**	41.7	4.5	41.3
**194**	8.3	2.6	9.2
**196**	3.0	2.6	3.2
**197**	5.6	2.6	4.4
**198**	3.0	2.7	1.7
**204**	-1.9	3.2	0.6
**207**	1.7	1.9	0.3
**208**	-0.2	2.3	0.2
**215**	2.9	2.1	1.4
**217**	6.2	1.7	5.3
**223**	3.0	0.5	2.3
**225**	5.6	1.8	4.7
**226**	3.7	2.0	0.9
**227**	-0.1	1.7	0.5
**231**	1.9	2.3	0.5
**232**	-1.5	2.6	0.3
**233**	3.1	3.2	0.1
**235**	-1.3	1.3	0.5
**240**	0.1	1.2	0.3
**243**	2.9	1.8	0.7
**244**	10.0	4.3	10.6
**246**	3.5	2.4	2.1
**250**	2.5	2.2	1.8
**259**	1.2	2.1	1.9
**260**	4.4	2.0	2.3
**263**	1.2	2.7	0.1
**264**	-0.8	2.1	0.3
**266**	1.4	0.6	0.1
**275**	-0.2	3.4	0.1
**279**	-1.2	1.2	0.6
**280**	4.1	2.2	1.0
**281**	3.0	1.6	2.1
**282**	53.9	3.7	54.3
**286**	-	-	452.5
**291**	1.0	3.1	0.9
**293**	13.4	3.0	15.4

**359**	3	4
**360**	28	31
**361**	13	12
**362**	0.5	-1
**366**	15	12.5
**368**	26.5	29.5
**369**	11.5	11.5
**370**	20.5	21.5
**371**	26.5	27.5
**377**	-4.5	-6.5
**379**	-41.5	-40
**383**	-33.5	-27.5
**386**	-13	-12
**387**	-14.5	-13.5
**388**	-20	-18
**392**	-6	-6.5
**394**	-3	-3.5
**395**	-5.5	-7
**401**	-9.5	-11.5
**402**	-2.5	-2.5
**403**	-4	-3
**413**	-3.5	-4
**414**	-13	-12
**416**	-12.5	-13
**419**	-9.5	-7.5
**430**	-10	-10.5
**431**	-6.5	-8.5
**435**	-6	-7.5
**438**	-7.5	-7
**439**	0	-3
**444**	-7	-7.5
**446**	-9	-9
**449**	-2.5	-3
**450**	-7	-5.5
**454**	-3	-3.5
**457**	-3.5	-3.5
**458**	-5.5	-6
**459**	-2.5	-2.5
**461**	-3.5	-4
**462**	-4	-4
**463**	-4.5	-6
**464**	-6	-6
**467**	-3.5	-4
**470**	-1	-2
**472**	-2	-3
**474**	-2	-3
**475**	-4	-4.5
**479**	-3	-3.5
**481**	-3.5	-3.5
**484**	-3	-3.5
**485**	-4	-4
**486**	-4	-4
**492**	-1	-2
**495**	-2	-2
**498**	-2	-2

**386**	5	7
**387**	8	8.5
**392**	5	4
**394**	4.5	3.5
**395**	11	6.5
**396**	0.5	3.5
**401**	13	11
**402**	3	3
**403**	2	2
**413**	0	-1
**414**	8	6
**416**	8	6
**419**	2.5	2
**430**	7	5.5
**431**	8.5	6.5
**435**	7.5	5.5
**438**	3	3.5
**439**	0	2
**444**	1.5	3.5
**445**	2.5	2.5
**446**	3	4
**449**	1	1
**454**	1.5	2
**457**	1.5	1.5
**458**	2.5	2.5
**459**	1.5	1
**461**	0	1
**462**	2	1.5
**463**	-0.5	1.5
**464**	2.5	2.5
**467**	-0.5	1
**470**	-0.5	0.5
**472**	1	1
**474**	1	1.5
**475**	1	2
**479**	1.5	1.5
**481**	1	1.5
**484**	0.5	1
**485**	1	1
**486**	0.5	1.5
**492**	0.5	0.5
**495**	0	0.5
**498**	0.5	0.5

**458**	1.5	1.5
**459**	-3	-3.5
**461**	1	-0.5
**462**	0.5	-1
**463**	-0.5	3
**464**	3.5	2
**467**	2	0.5
**470**	-1	-2
**472**	-2.5	-1
**474**	-3.5	-1.5
**475**	1	0.5
**479**	-1	-2
**481**	1	-1.5
**484**	-0.5	-1.5
**485**	1	0
**486**	0	-0.5
**492**	-1	-2.5
**495**	6	-3
**498**	2.5	-2.5

**299**	3.6	2.3	1.2
**307**	8.0	1.3	8.2
**310**	-	-	13.7
**312**	4.9	2.1	3.2
**314**	3.6	5.5	0.8
**316**	0.7	1.7	0.2
**317**	1.2	2.7	1.4
**318**	0.7	2.0	0.2
**320**	4.0	2.1	0.5
**321**	2.2	3.1	1.4
**327**	2.3	0.7	0.9
**328**	3.6	5.9	3.1
**330**	5.1	2.3	7.0
**332**	18.8	3.6	15.8
**333**	-	-	264.3
**338**	4.0	3.3	4.3
**339**	2.5	8.5	2.6
**345**	0.4	4.4	0.5
**349**	9.3	5.9	9.0
**352**	10.4	3.7	12.1
**354**	29.7	5.8	29.6
**355**	-	-	278.5
**356**	49.0	2.7	48.4
**358**	20.5	3.3	21.1
**359**	4.6	5.4	5.5
**360**	3.4	1.9	2.6
**361**	4.3	1.0	2.2
**362**	2.8	1.8	1.7
**366**	2.9	2.5	0.9
**368**	-0.1	2.4	1.5
**369**	1.7	2.5	0.4
**370**	3.7	1.6	0.7
**371**	3.7	2.1	1.9
**377**	3.0	1.5	4.2
**379**	9.1	2.4	8.5
**381**	32.2	6.6	30.2
**383**	3.9	1.3	3.3
**385**	5.3	6.7	2.9
**386**	1.7	2.2	0.6
**387**	3.5	0.9	1.2
**388**	4.1	3.9	2.0
**392**	2.2	1.4	0.3
**394**	2.4	1.2	0.2
**395**	1.3	2.2	0.3
**396**	2.5	9.4	0.2
**401**	3.6	1.1	0.8
**402**	-1.3	1.7	0.3
**403**	3.2	1.1	0.5
**413**	1.0	1.9	0.8
**414**	2.4	1.6	0.9
**416**	0.6	2.2	0.8
**419**	2.1	3.0	0.8
**423**	7.4	1.4	6.6
**424**	3.2	1.9	2.5
**430**	2.9	1.3	0.5
**431**	-1.7	1.2	0.4
**435**	3.8	1.2	0.3
**438**	1.0	1.5	0.3
**439**	-1.1	1.2	0.1
**444**	-0.4	2.5	0.3
**445**	0.1	0.9	0.4

	**446**	-0.2	1.9	0.3
	**449**	1.0	1.1	0.1
	**450**	-	-	0.2
	**454**	-0.3	2.0	0.0
	**457**	-0.5	1.5	0.0
	**458**	0.8	1.2	0.1
	**459**	3.0	2.5	0.1
	**461**	0.1	2.2	0.1
	**462**	2.1	1.5	0.1
	**463**	-0.8	2.8	0.1
	**464**	1.4	2.2	0.1
	**467**	1.3	1.5	0.1
	**470**	0.9	2.8	0.0
	**472**	0.8	2.4	0.1
	**474**	2.9	2.5	0.1
	**475**	1.2	1.7	0.1
	**479**	1.7	1.7	0.0
	**481**	2.3	1.3	0.0
	**484**	3.6	2.5	0.0
	**485**	0.9	2.5	0.1
	**486**	0.9	3.1	0.1
	**492**	0.0	0.3	0.0
	**495**	-0.2	0.3	0.0
	**498**	-0.1	0.6	0.1

Table S5 Final magnetic susceptibility tensors from proton PCS and metal coordinates obtained from PREs.

Tensor	ΔX_{ax} (10^{-32} m^3)	ΔX_{rh} (10^{-32} m^3)	Paramagnetic centre (Å)			Orientation of principal axes (°)		
			X	Y	Z	α	β	γ
Individual fit								
Tb^{3+}	8.71 (±0.08)	0.57 (±0.07)	23.56 (±0.07)	33.10 (±0.09)	40.70 (±0.11)	169.22 (±0.19)	90.81 (±0.23)	110.71 (±2.64)
Tm^{3+}	-1.57 (±0.01)	-0.43 (±0.01)	23.95 (±0.07)	33.30 (±0.08)	39.27 (±0.10)	168.83 (±0.33)	87.97 (±0.26)	117.72 (±0.57)
Eu^{3+}	-0.71 (±0.01)	-0.25 (±0.01)	24.36 (±0.07)	33.41 (±0.11)	40.53 (±0.09)	172.07 (±0.28)	92.23 (±0.39)	18.84 (±0.65)
Ce^{3+}	1.12 (±0.01)	0.04 (±0.01)	24.64 (±0.06)	33.24 (±0.07)	40.43 (±0.08)	171.76 (±0.23)	87.75 (±0.25)	112.92 (±6.02)
Combined fit								
Tb^{3+}	8.92 (±0.07)	0.53 (±0.06)	23.99 (±0.04)	33.17 (±0.04)	40.46 (±0.06)	169.72 (±0.16)	91.14 (±0.23)	114.30 (±2.36)
Tm^{3+}	-1.52 (±0.01)	-0.35 (±0.01)	23.99 (±0.04)	33.17 (±0.04)	40.46 (±0.06)	167.89 (±0.27)	90.23 (±0.21)	117.18 (±0.81)
Eu^{3+}	-0.70 (±0.01)	-0.25 (±0.01)	23.99 (±0.04)	33.17 (±0.05)	40.46 (±0.06)	171.26 (±0.31)	91.04 (±0.38)	18.06 (±0.67)
Ce^{3+}	1.09 (±0.01)	0.02 (±0.02)	23.99 (±0.04)	33.17 (±0.05)	40.46 (±0.06)	170.10 (±0.25)	86.78 (±0.28)	101.11 (±15.63)
PREs (combined fit)								
			23.99 (±0.14)	32.38 (±0.14)	40.94 (±0.18)			
Position Mg^{2+} described in pdb 4M2A (UDP-Glc bound or also called post-reactive state)								
			26.66	36.37	40.68			

Results of two different fits are listed. *Individual* indicates a fitting process where all parameters were free and the tensors were calculated independently for each metal ion. *Combined* indicates a fitting process where all parameters were free but tensors were calculated simultaneously for all metal ions. For comparison, the position of the Mg^{2+} ion after PREs fitting and in the crystal structure of the post-reactive state (metal release) is shown at the end of the table.

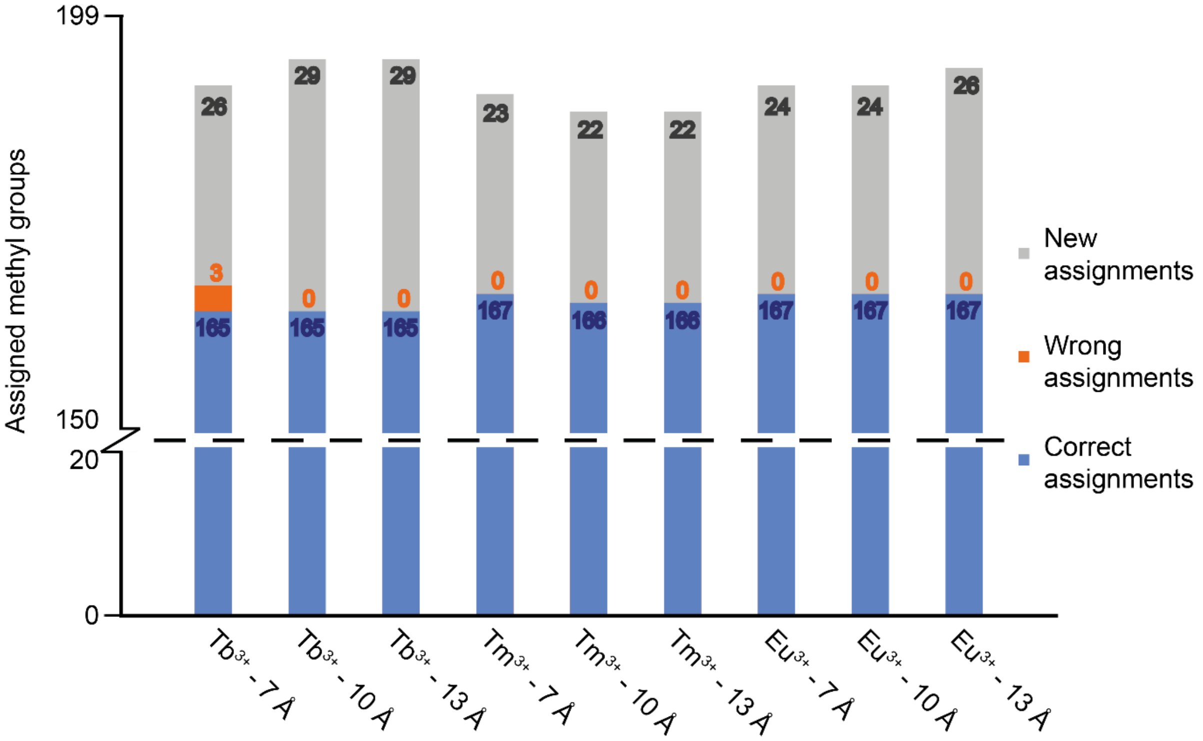

Fig S8 Determination of optimal distance for *MAP-XSII* calculations including PCS. Calculations were performed as described in Fig. S3. Blue and orange correspond to signals consistently assigned to the same residue in 20 MMC trials which matched or deviated from the NOE-based assignment, respectively. Gray indicates new assignments obtained exclusively through PCS. Three cut-off distances of 7, 10 and 13 Å were explored for each lanthanoid ion (Tb^{3+}, Tm^{3+} and Eu^{3+}).

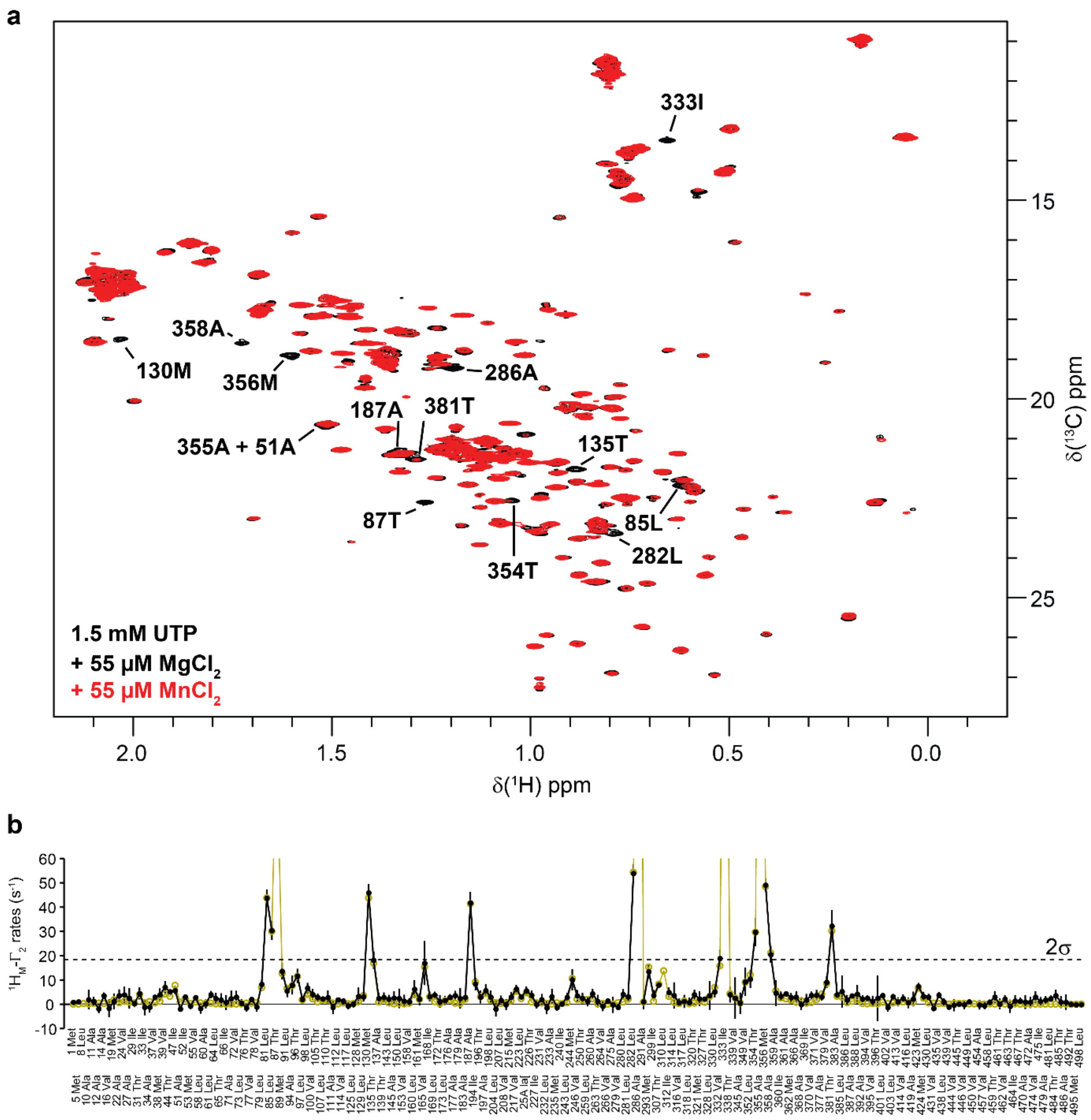

Fig S9 Analysis of PREs. **a** Superimposition of ^{1}H,^{13}C HMQC spectra of MIL^{proS}V^{proS}AT LmUGP at 200 µM concentration in the presence of 1.5 mM UTP and 55 µM of $MgCl_2$ (black) or $MnCl_2$ (red). Indicated are amino acids with methyl groups exhibiting PREs > 2σ. **b** Observed (solid black circles) and calculated (apo gold symbols) PREs for observable methyl groups of MIL^{proS}V^{proS}AT labeled LmUGP. Errors in observed PREs correspond to one σ. Dashed line corresponds to 2σ from all observed PREs.

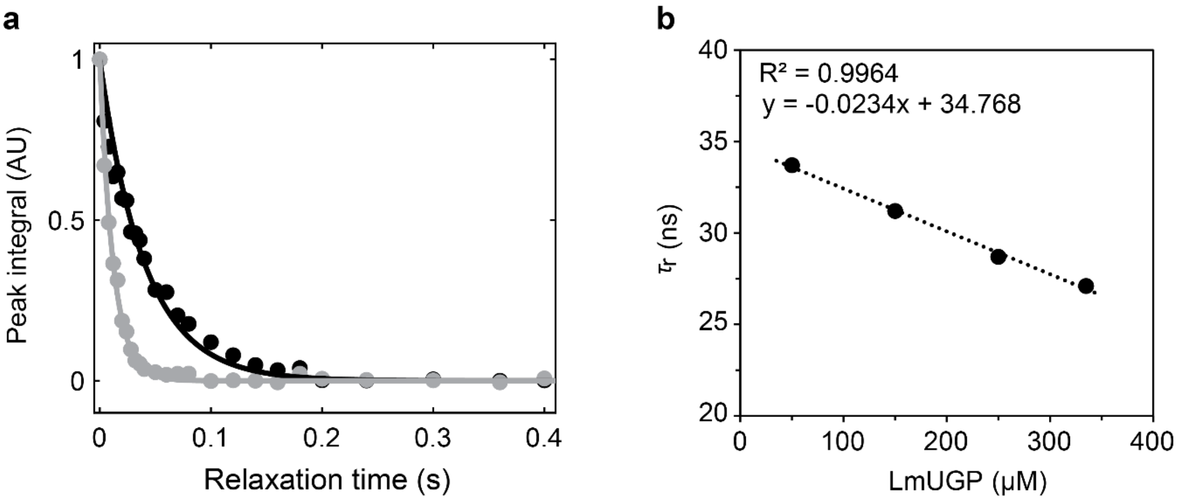

Fig. S10 TRACT is used to estimate protein rotational correlation times τ_r. This value is required to calculate PREs, and to find optimal sample conditions for 4D HMQC-NOESY-HMQC experiments. **a** Decay curves at lowest LmUGP concentration (50 µM). Black and grey curves correspond to ^{15}N R_α and R_β, respectively. **b** Protein dilution series reveals a decrease in τ_r associated to protein concentration. The linear equation obtained form the fitting of the corresponding τ_r can be used to estimate τ_r at any given protein concentration. Experiments acquired at 500 MHz and 273 K.

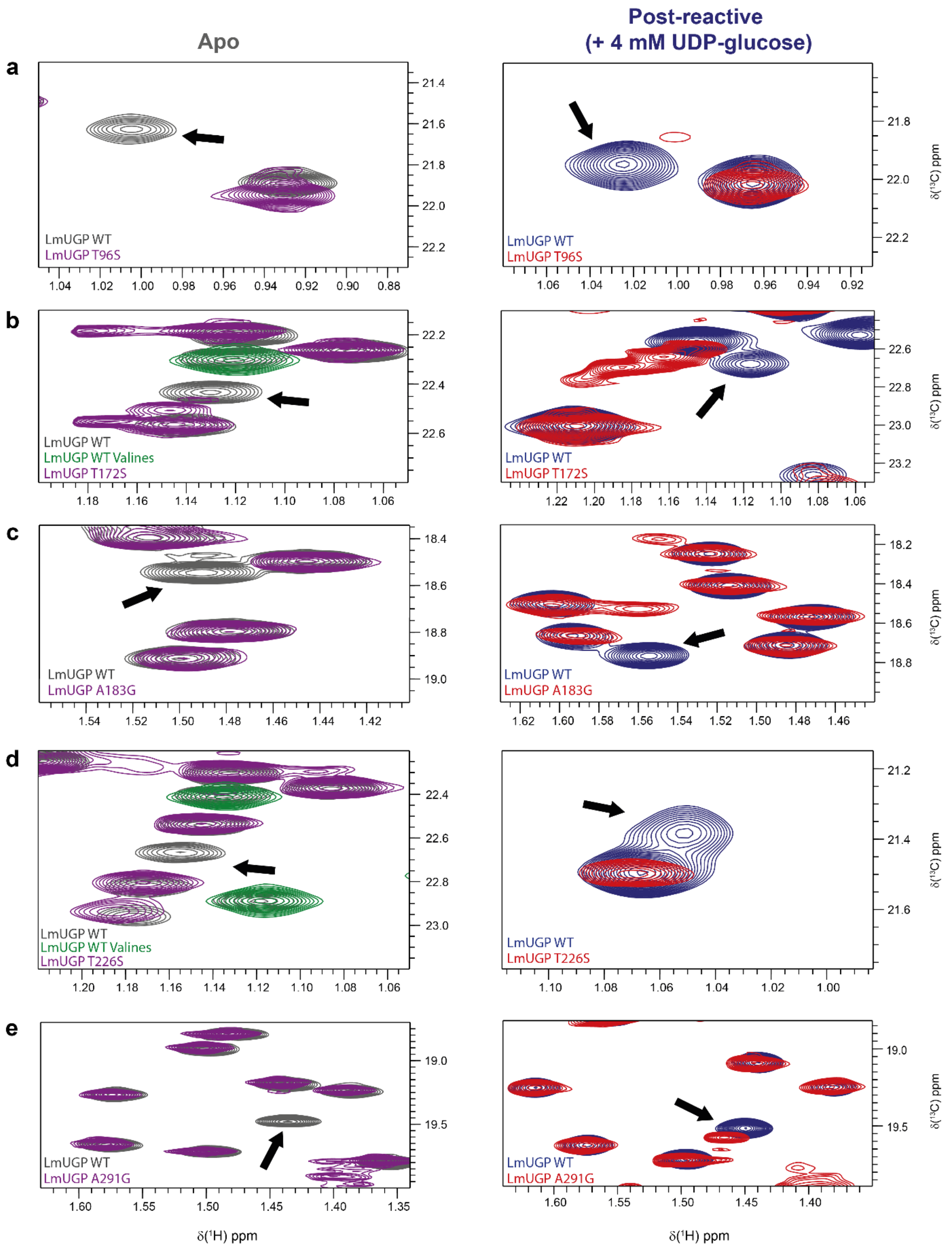

Fig. S11 Assignments obtained *via* mutagenesis for **a** T96, **b** T172, **c** A183, **d** T226 and **e** A291. Superimposition of ^{1}H,^{13}C HMQC spectra of MIL^{proS}V^{proS}AT LmUGP. (*Left panels*) Wild type and single-point mutants in the apo state are shown in grey and violet, respectively. Certain peaks from Thr and Ala appear at similar spectral frequencies. When signals from both amino acid types are in the same spectrum, Ala from the wild type are indicated in green. (*Right panels*) Wild type and single-point mutants in the UDP-Glc bound state (+ 4 mM UDP-glucose) are colored in red and blue, respectively. Arrows indicate the missing signal of the corresponding mutant. Spectra were acquired at 600 MHz and 293 K.

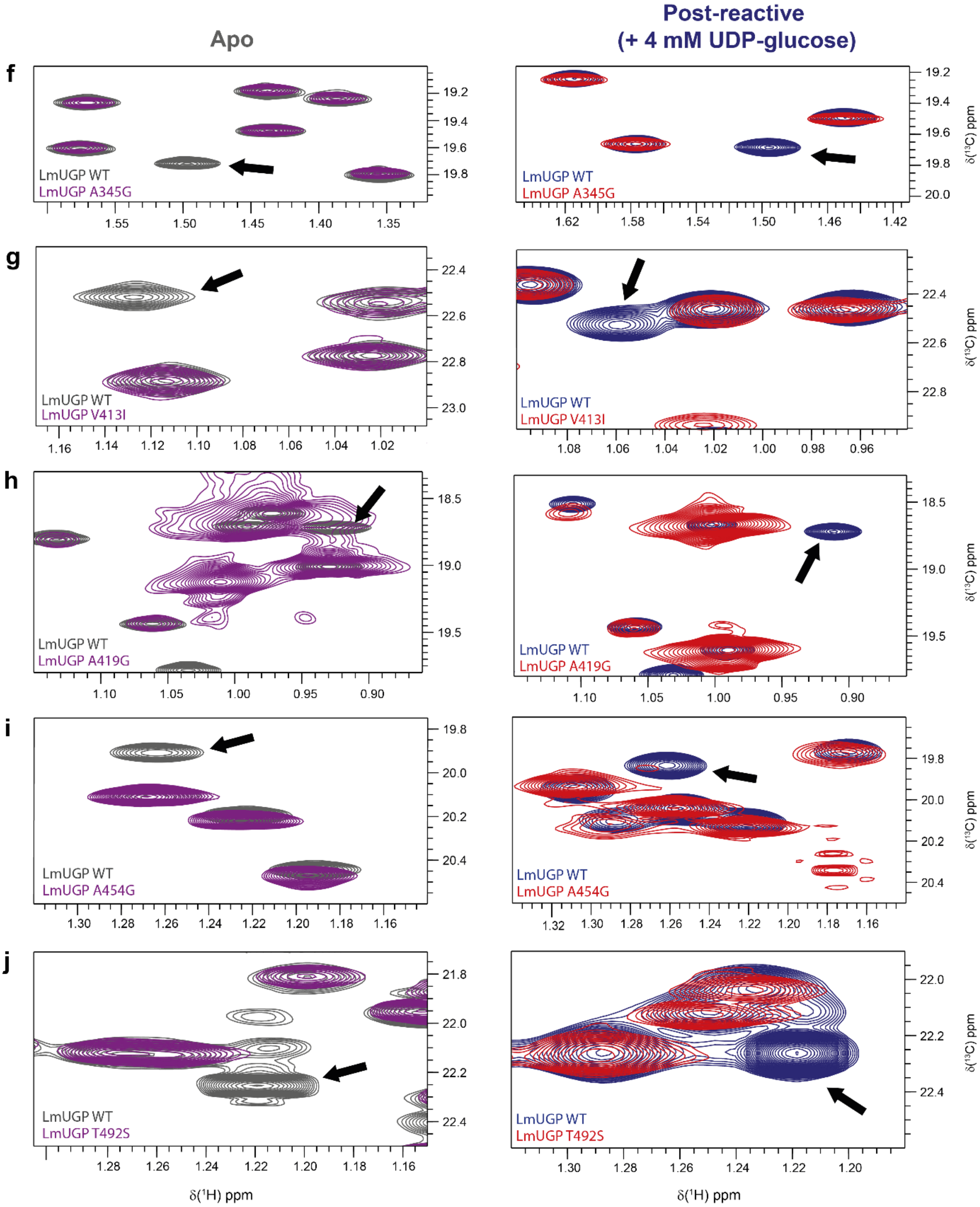

Fig. S11 (continuation): Assignments obtained *via* mutagenesis for **f** A345, **g** V413, **h** A419, **i** A454 and **j** T492.

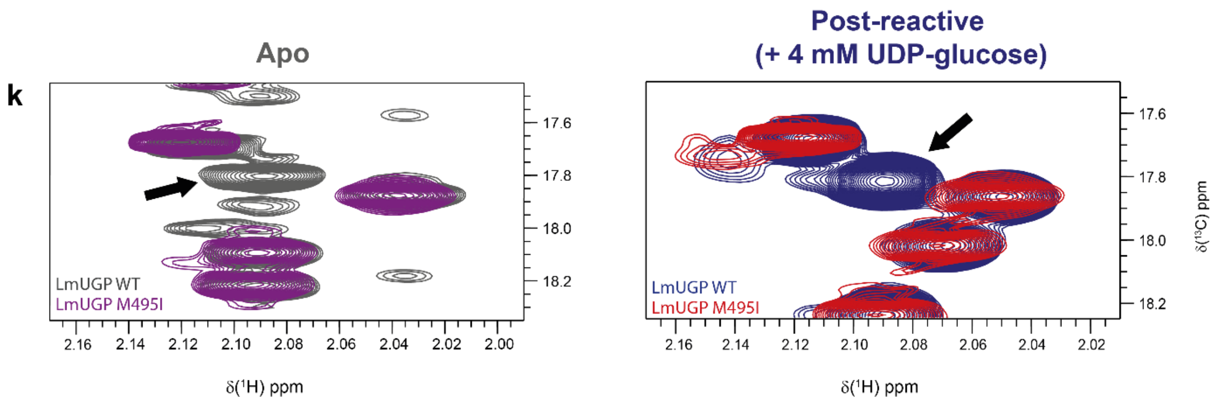

Fig. S11 (continuation): Assignment obtained *via* mutagenesis for **k** M495.

Table S6 ^{1}H and ^{13}C chemical shifts of the apo and UDP-Glc bound conformations of MIL^{proS}V^{proS}AT methyl-labeled LmUGP.

			Apo		UDP-Glc bound	
**Amino acid number**	**Amino acid type**	**^{13}C-labeled methyl group**	**δ^{1}H (ppm)**	**δ^{13}C (ppm)**	**δ^{1}H (ppm)**	**δ^{13}C (ppm)**
1	MET	Cε	2.091	18.218	2.089	18.239
5	MET	Cε	2.116	17.685	2.114	17.683
8	LEU	Cδ2	0.767	23.356	0.769	23.329
10	ALA	Cβ	1.513	18.406	1.513	18.412
11	ALA	Cβ	1.575	19.671	1.577	19.697
12	ALA	Cβ	1.386	19.251	1.380	19.243
14	ALA	Cβ	1.610	18.494	1.603	18.499
16	VAL	Cγ2	0.992	24.056	0.992	24.121
19	MET	Cε	2.044	21.168	2.018	20.988
22	ALA	Cβ	1.377	20.141	1.288	20.105
24	VAL	Cγ2	1.113	23.899	1.093	23.736
27	ALA	Cβ	1.710	18.683	1.592	18.660
29	ILE	Cδ1	0.845	14.906	0.839	14.962
31	THR	Cγ2	1.274	22.131	1.290	22.263
33	ILE	Cδ1	0.794	12.153	0.799	12.336
34	ALA	Cβ	1.517	18.375	1.524	18.248
37	VAL	Cγ2	1.142	23.564	1.149	23.546
38	MET	Cε	2.035	17.881	2.049	17.865
39	VAL	Cγ2	0.719	23.416	0.704	23.088
44	THR	Cγ2	1.150	21.967	1.169	21.766
47	ILE	Cδ1	0.770	14.976	0.766	14.973
51	ALA	Cβ	1.527	21.499	1.524	21.407
52	ILE	Cδ1	0.507	14.001	0.518	13.847
53	MET	Cε	2.091	17.807	2.068	18.019
55	VAL	Cγ2	0.952	22.537	0.899	22.709
58	LEU	Cδ2	0.769	22.521	0.763	22.643
60	ALA	Cβ	1.393	21.749	1.432	22.091
61	LEU	Cδ2	0.732	26.668	0.768	26.727
64	LEU	Cδ2	0.637	22.293	0.610	22.410
65	THR	Cγ2	1.084	22.379	1.094	22.362
66	ILE	Cδ1	0.811	12.703	0.819	12.760
71	ALA	Cβ	1.480	18.802	1.484	18.711
72	VAL	Cγ2	1.128	24.568	1.152	24.330
73	LEU	Cδ2	0.882	24.977	0.903	25.705
76	THR	Cγ2	1.203	21.480	1.229	21.676
77	VAL	Cγ2	0.619	23.168	0.577	23.104
78	VAL	Cγ2	1.119	22.947	1.174	23.433

79	LEU	Cδ2	0.635	26.584	0.581	26.759
81	LEU	Cδ2	0.755	23.624	0.805	23.762
85	LEU	Cδ2	0.568	23.119	0.611	23.449
87	THR	Cγ2	1.288	23.257	1.266	23.220
89	MET	Cε	1.837	17.620	1.836	17.150
91	LEU	Cδ2	0.836	26.766	0.909	24.939
94	ALA	Cβ	1.250	18.814	1.264	18.981
96	THR	Cγ2	1.007	21.649	1.025	21.948
97	LEU	Cδ2	-0.017	22.976	0.280	23.622
98	LEU	Cδ2	0.708	26.060	0.796	27.682
100	VAL	Cγ2	0.182	20.600	0.138	21.767
105	THR	Cγ2	1.176	23.072	1.209	23.030
107	LEU	Cδ2	0.791	25.655	0.763	24.789
110	THR	Cγ2	0.835	24.901	1.083	23.263
111	ALA	Cβ	0.514	16.884	0.432	16.938
112	LEU	Cδ2	0.669	22.987	0.699	22.656
114	VAL	Cγ2	0.722	22.809	0.390	22.894
117	LEU	Cδ2	1.113	23.900	1.120	24.064
125	LEU	Cδ2	1.010	29.119	1.013	27.853
128	MET	Cε	1.650	16.909	1.649	16.913
129	LEU	Cδ2	0.838	25.669	0.896	25.967
130	MET	Cε	1.903	18.903	2.080	19.464
135	THR	Cγ2	0.877	22.927	0.889	22.631
137	ALA	Cβ	1.446	18.499	1.471	18.568
139	THR	Cγ2	1.199	21.818	1.237	21.681
143	LEU	Cδ2	0.970	24.057	0.944	23.793
145	ALA	Cβ	1.264	20.022	1.254	20.043
150	LEU	Cδ2	0.802	25.391	0.810	25.463
153	VAL	Cγ2	0.971	18.626	1.003	18.667
158	VAL	Cγ2	1.019	22.545	1.117	22.680
160	LEU	Cδ2	0.683	27.023	0.627	27.575
161	MET	Cε	1.824	16.343	1.955	17.243
165	VAL	Cγ2	0.930	19.015	0.991	19.606
168	ILE	Cδ1	0.794	15.173	0.795	15.227
169	LEU	Cδ2	0.907	22.997	0.906	22.931
172	THR	Cγ2	1.144	22.547	1.145	22.560
173	LEU	Cδ2	0.824	22.587	0.824	22.653
176	ALA	Cβ	0.974	20.724	0.980	20.586
177	ALA	Cβ	1.346	22.318	1.341	22.264
179	ALA	Cβ	1.189	18.748	1.191	18.761
183	ALA	Cβ	1.489	18.550	1.555	18.766
187	ALA	Cβ	1.337	22.779	1.376	22.296
194	ILE	Cδ1	0.558	16.322	0.595	15.457

196	THR	Cγ2	1.172	22.934	1.209	23.030
197	ALA	Cβ	1.359	18.341	1.350	18.275
198	LEU	Cδ2	0.574	24.790	0.551	24.634
204	LEU	Cδ2	1.009	28.072	0.994	27.983
207	LEU	Cδ2	0.443	23.749	0.474	23.406
208	VAL	Cγ2	0.936	24.891	0.924	24.895
215	MET	Cε	1.727	17.864	1.705	17.861
217	VAL	Cγ2	0.706	22.685	0.650	23.074
223	LEU	Cδ2	0.813	25.455	1.092	23.736
225	ALA	Cβ	1.500	18.915	1.566	21.118
226	THR	Cγ2	1.155	22.697	1.049	21.358
227	ILE	Cδ1	0.880	13.044	0.856	13.699
231	VAL	Cγ2	0.384	23.178	0.493	23.420
232	LEU	Cδ2	0.413	23.653	0.319	23.819
233	ALA	Cβ	1.265	18.583	1.291	18.650
235	MET	Cε	1.807	17.275	1.784	17.167
240	ILE	Cδ1	0.496	14.902	0.430	14.413
243	LEU	Cδ2	0.565	27.712	0.589	27.579
244	MET	Cε	2.072	18.902	1.999	18.512
246	VAL	Cγ2	0.294	19.964	0.558	20.608
250	THR	Cγ2	1.357	22.314	1.233	22.032
259	LEU	Cδ2	0.818	27.833	0.823	28.043
260	ALA	Cβ	1.477	24.436	1.519	24.198
263	THR	Cγ2	0.848	23.020	0.854	23.048
264	VAL	Cγ2	0.810	21.106	0.806	20.987
266	VAL	Cγ2	0.818	21.085	0.817	21.040
275	ALA	Cβ	1.351	22.690	1.350	22.680
279	VAL	Cγ2	0.873	21.110	0.870	20.785
280	LEU	Cδ2	0.907	24.335	0.892	23.781
281	LEU	Cδ2	0.639	27.248	0.610	27.296
282	LEU	Cδ2	0.794	25.530	0.647	27.762
286	ALA	Cβ	1.357	19.799	1.307	19.946
291	ALA	Cβ	1.436	19.484	1.450	19.516
293	MET	Cε	1.879	16.983	1.977	17.148
299	ILE	Cδ1	0.736	14.588	0.707	14.817
307	THR	Cγ2	0.893	23.513	0.756	22.610
310	LEU	Cδ2	1.085	25.162	1.026	25.848
312	ILE	Cδ1	0.896	16.253	0.863	16.066
314	LEU	Cδ2	0.849	22.721	0.805	22.350
316	VAL	Cγ2	1.102	23.516	1.093	23.736
317	LEU	Cδ2	1.159	27.009	1.162	27.007
318	LEU	Cδ2	0.996	27.159	0.990	27.089
320	THR	Cγ2	0.888	21.007	0.901	21.085

321	MET	Cε	1.594	16.774	1.542	16.714
327	THR	Cγ2	0.930	21.926	0.965	22.012
328	LEU	Cδ2	0.282	26.175	0.214	25.464
330	LEU	Cδ2	0.658	22.991	0.637	23.074
332	VAL	Cγ2	0.797	24.109	0.720	24.094
333	ILE	Cδ1	0.672	14.111	0.607	14.767
338	THR	Cγ2	0.959	23.107	0.955	23.077
339	VAL	Cγ2	0.617	23.211	0.609	23.230
345	ALA	Cβ	1.497	19.723	1.496	19.721
349	VAL	Cγ2	0.240	18.669	0.222	18.684
352	LEU	Cδ2	0.504	24.257	0.497	24.509
354	THR	Cγ2	1.083	23.732	1.003	23.525
355	ALA	Cβ	1.524	21.401	1.310	20.786
356	MET	Cε	1.651	19.571	1.669	18.475
358	ALA	Cβ	1.776	19.816	1.753	19.775
359	ALA	Cβ	1.294	19.273	1.211	18.646
360	ILE	Cδ1	0.951	16.671	0.957	16.859
361	ALA	Cβ	1.434	20.558	1.457	20.952
362	MET	Cε	1.655	18.260	1.650	17.948
366	ALA	Cβ	1.767	24.203	1.746	24.362
368	ALA	Cβ	1.208	24.107	1.251	24.560
369	ILE	Cδ1	0.086	14.219	0.188	14.247
370	VAL	Cγ2	0.677	23.924	0.892	24.029
371	VAL	Cγ2	0.989	18.718	1.053	17.857
377	ALA	Cβ	1.192	20.368	1.170	19.766
379	VAL	Cγ2	0.967	23.149	1.019	23.014
381	THR	Cγ2	1.331	22.096	1.284	22.263
383	ALA	Cβ	1.438	19.187	1.440	19.077
385	LEU	Cδ2	0.955	26.878	1.006	24.768
386	LEU	Cδ2	0.956	26.880	0.970	26.919
387	ALA	Cβ	1.571	19.278	1.615	19.246
388	LEU	Cδ2	-0.181	22.374	0.214	25.134
392	ALA	Cβ	1.133	18.807	1.106	18.514
394	VAL	Cγ2	0.698	20.025	0.668	19.541
395	VAL	Cγ2	1.022	22.774	0.914	22.452
396	THR	Cγ2	1.111	23.987	1.085	24.132
401	LEU	Cδ2	0.245	27.015	0.111	25.448
402	VAL	Cγ2	0.599	19.919	0.599	20.145
403	LEU	Cδ2	0.167	23.897	0.182	23.929
413	VAL	Cγ2	1.134	22.409	1.058	22.528
414	VAL	Cγ2	0.818	20.464	0.786	20.315
416	LEU	Cδ2	0.629	23.659	0.611	23.449
419	ALA	Cβ	0.926	18.729	0.911	18.720

423	MET	Cε	2.102	19.209	2.119	19.307
424	MET	Cε	2.103	17.433	2.142	17.747
430	LEU	Cδ2	0.435	26.943	0.436	26.830
431	VAL	Cγ2	0.378	18.159	0.344	18.249
435	VAL	Cγ2	0.974	22.516	0.963	22.456
438	LEU	Cδ2	0.721	25.633	0.719	25.576
439	VAL	Cγ2	0.798	23.263	0.780	23.174
444	VAL	Cγ2	0.774	22.608	0.763	22.643
445	THR	Cγ2	1.253	22.138	1.249	22.113
446	VAL	Cγ2	0.747	21.621	0.743	21.653
449	LEU	Cδ2	0.588	25.419	0.562	25.191
450	VAL	Cγ2	0.767	23.356	0.769	23.329
454	ALA	Cβ	1.263	19.834	1.261	19.834
457	VAL	Cγ2	0.888	20.745	0.881	20.738
458	LEU	Cδ2	0.902	27.081	0.897	27.054
459	THR	Cγ2	1.025	22.464	1.020	22.461
461	THR	Cγ2	1.131	22.315	1.130	22.311
462	VAL	Cγ2	0.995	23.349	0.989	23.361
463	THR	Cγ2	1.124	21.965	1.119	21.969
464	ILE	Cδ1	0.756	15.817	0.754	15.794
467	THR	Cγ2	1.131	21.876	1.127	21.903
470	ALA	Cβ	1.438	20.690	1.433	20.596
472	ALA	Cβ	1.060	19.440	1.060	19.432
474	VAL	Cγ2	0.794	21.365	0.793	21.362
475	ILE	Cδ1	0.173	11.845	0.185	11.851
479	ALA	Cβ	1.221	20.110	1.220	20.111
481	LEU	Cδ2	0.838	25.033	0.834	25.042
484	THR	Cγ2	1.034	19.787	1.031	19.791
485	THR	Cγ2	1.071	21.490	1.067	21.491
486	ALA	Cβ	1.494	22.205	1.493	22.178
492	THR	Cγ2	1.218	22.260	1.218	22.263
495	MET	Cε	2.090	18.096	2.090	17.815
498	LEU	Cδ2	0.842	24.063	0.840	24.067

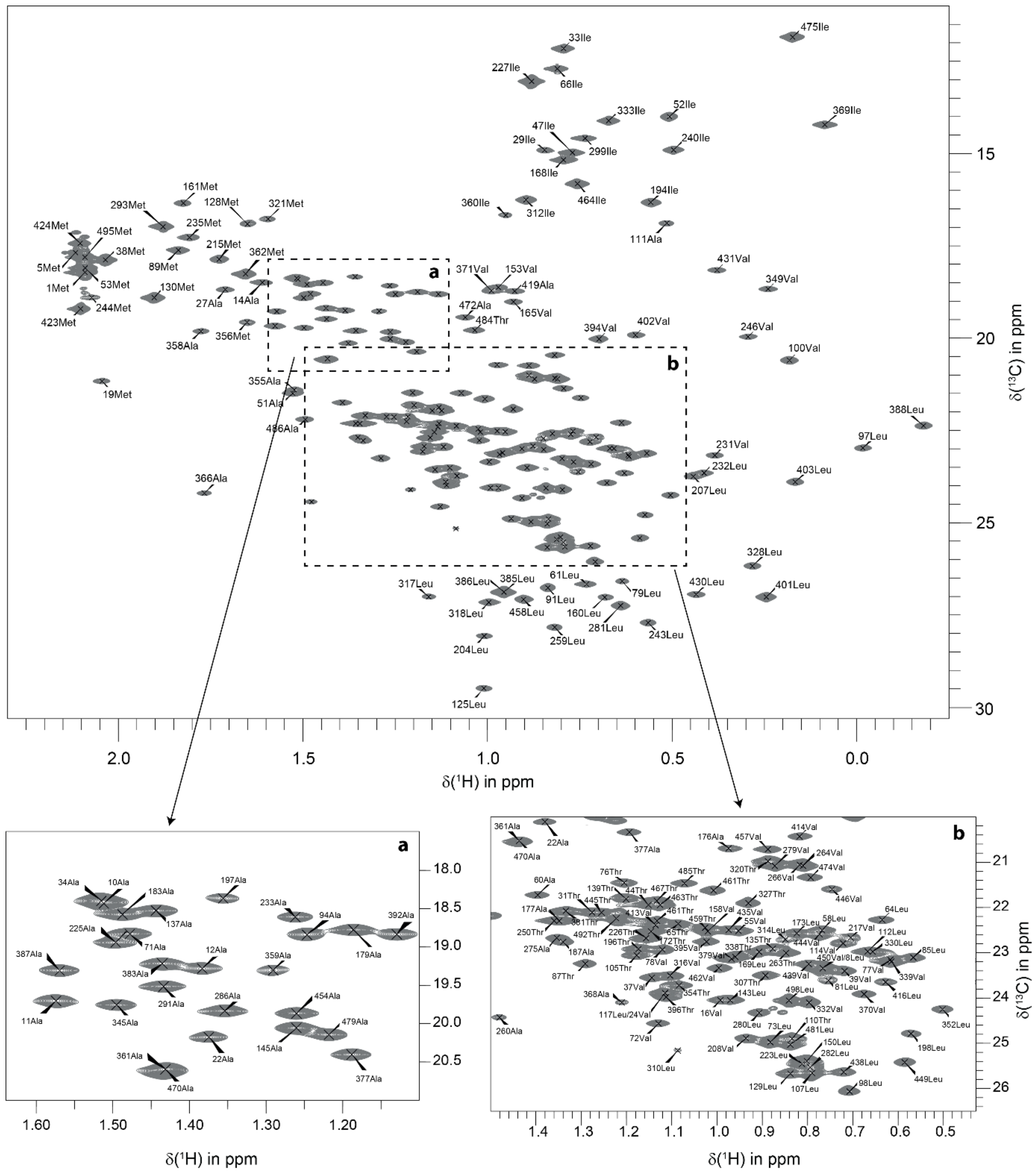

Fig. S12 Complete assignment of the apo state of MIL^{proS}V^{proS}AT LmUGP. Due to signal crowding, the sections **a** and **b** located in the centre of the spectrum are reproduced in the lower panels. ^{1}H,^{13}C HMQC spectrum was acquired from a sample containing 450 µM protein at 293 K and 900 MHz.

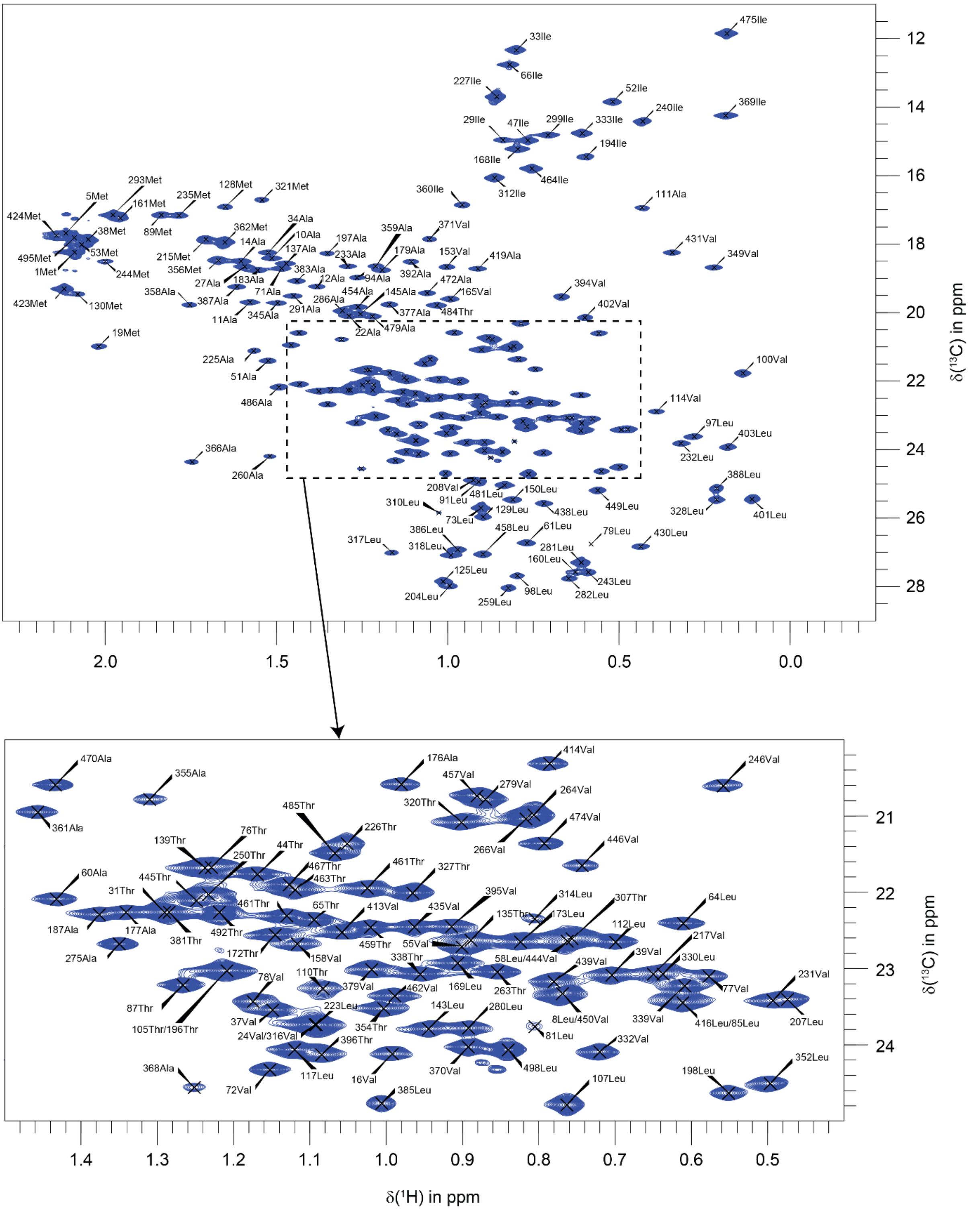

Fig. S13 Complete assignment of the UDP-Glc bound conformation of MIL^{proS}V^{proS}AT LmUGP. Due to signal crowding, the section corresponding to the middle of the spectrum is separately reproduced in the lower panel. ^{1}H,^{13}C HMQC spectrum was acquired from a sample containing 450 µM protein and 12 mM UDP-glucose at 293 K and 950 MHz.

References:

1. Muller-Hermes, C., Creutznacher, R. & Mallagaray, A. Complete assignment of Ala, Ile, Leu(ProS), Met and Val(ProS) methyl groups of the protruding domain from human norovirus GII.4 Saga. *Biomol NMR Assign* **14**, 123-130 (2020).
2. Schütz, S. & Sprangers, R. Methyl TROSY spectroscopy: A versatile NMR approach to study challenging biological systems. *Progress in Nuclear Magnetic Resonance Spectroscopy* (2019).
3. Gelis, I. et al. Structural basis for signal-sequence recognition by the translocase motor SecA as determined by NMR. *Cell* **131**, 756-69 (2007).
4. Gardner, K.H. & Kay, L.E. Production and Incorporation of 15N, 13C, 2H (1H-δ1 Methyl) Isoleucine into Proteins for Multidimensional NMR Studies. *Journal of the American Chemical Society* **119**, 7599-7600 (1997).
5. Gans, P. et al. Stereospecific Isotopic Labeling of Methyl Groups for NMR Spectroscopic Studies of High-Molecular-Weight Proteins. *Angewandte Chemie International Edition* **49**, 1958-1962 (2010).
6. Mas, G., Crublet, E., Hamelin, O., Gans, P. & Boisbouvier, J. Specific labeling and assignment strategies of valine methyl groups for NMR studies of high molecular weight proteins. *J Biomol NMR* **57**, 251-62 (2013).
7. Flugge, F. & Peters, T. Complete assignment of Ala, Ile, Leu, Met and Val methyl groups of human blood group A and B glycosyltransferases using lanthanide-induced pseudocontact shifts and methyl-methyl NOESY. *J Biomol NMR* **70**, 245-259 (2018).
8. Velyvis, A., Ruschak, A.M. & Kay, L.E. An economical method for production of (2)H, (13)CH3-threonine for solution NMR studies of large protein complexes: application to the 670 kDa proteasome. *PLoS One* **7**, e43725 (2012).
9. Führing, J. et al. Catalytic Mechanism and Allosteric Regulation of UDP-Glucose Pyrophosphorylase from Leishmania major. *ACS Catalysis* **3**, 2976-2985 (2013).
10. Fiser, A. & Sali, A. ModLoop: automated modeling of loops in protein structures. *Bioinformatics* **19**, 2500-1 (2003).
11. Arai, M., Ferreon, J.C. & Wright, P.E. Quantitative Analysis of Multisite Protein–Ligand Interactions by NMR: Binding of Intrinsically Disordered p53 Transactivation Subdomains with the TAZ2 Domain of CBP. *Journal of the American Chemical Society* **134**, 3792-3803 (2012).
